# Systems-level analysis of age-related macular degeneration reveals global biomarkers and phenotype-specific functional networks

**DOI:** 10.1186/gm315

**Published:** 2012-02-24

**Authors:** Aaron M Newman, Natasha B Gallo, Lisa S Hancox, Norma J Miller, Carolyn M Radeke, Michelle A Maloney, James B Cooper, Gregory S Hageman, Don H Anderson, Lincoln V Johnson, Monte J Radeke

**Affiliations:** 1 Center for the Study of Macular Degeneration, Neuroscience Research Institute, Biological Sciences 2 Building, University of California, Santa Barbara, CA 93106-5060, USA; 2 Department of Ophthalmology and Visual Sciences, University of Iowa, 200 Hawkins Drive Iowa City, IA 52242-1109, USA; 3 Department of Ophthalmology and Visual Sciences, John A Moran Eye Center, University of Utah, 65 Mario Capecchi Drive, Salt Lake City, UT 84132-5230, USA; 4 Molecular, Cellular, and Developmental Biology Department, Life Sciences Building, University of California, Santa Barbara, CA 93106-9610, USA; 5 Current address: Institute for Stem Cell Biology and Regenerative Medicine, Stanford University School of Medicine, 265 Campus Drive, Stanford, CA 94305, USA

## Abstract

**Background:**

Age-related macular degeneration (AMD) is a leading cause of blindness that affects the central region of the retinal pigmented epithelium (RPE), choroid, and neural retina. Initially characterized by an accumulation of sub-RPE deposits, AMD leads to progressive retinal degeneration, and in advanced cases, irreversible vision loss. Although genetic analysis, animal models, and cell culture systems have yielded important insights into AMD, the molecular pathways underlying AMD's onset and progression remain poorly delineated. We sought to better understand the molecular underpinnings of this devastating disease by performing the first comparative transcriptome analysis of AMD and normal human donor eyes.

**Methods:**

RPE-choroid and retina tissue samples were obtained from a common cohort of 31 normal, 26 AMD, and 11 potential pre-AMD human donor eyes. Transcriptome profiles were generated for macular and extramacular regions, and statistical and bioinformatic methods were employed to identify disease-associated gene signatures and functionally enriched protein association networks. Selected genes of high significance were validated using an independent donor cohort.

**Results:**

We identified over 50 annotated genes enriched in cell-mediated immune responses that are globally over-expressed in RPE-choroid AMD phenotypes. Using a machine learning model and a second donor cohort, we show that the top 20 global genes are predictive of AMD clinical diagnosis. We also discovered functionally enriched gene sets in the RPE-choroid that delineate the advanced AMD phenotypes, neovascular AMD and geographic atrophy. Moreover, we identified a graded increase of transcript levels in the retina related to wound response, complement cascade, and neurogenesis that strongly correlates with decreased levels of phototransduction transcripts and increased AMD severity. Based on our findings, we assembled protein-protein interactomes that highlight functional networks likely to be involved in AMD pathogenesis.

**Conclusions:**

We discovered new global biomarkers and gene expression signatures of AMD. These results are consistent with a model whereby cell-based inflammatory responses represent a central feature of AMD etiology, and depending on genetics, environment, or stochastic factors, may give rise to the advanced AMD phenotypes characterized by angiogenesis and/or cell death. Genes regulating these immunological activities, along with numerous other genes identified here, represent promising new targets for AMD-directed therapeutics and diagnostics.

**Please see related commentary: http://www.biomedcentral.com/1741-7015/10/21/abstract:**

## Background

The neural retina, retinal pigmented epithelium (RPE), and choroid tissue complex is one of the most physiologically active tissues in humans and arguably our most important sensory organ [1]. Perhaps due to its high metabolic rate, unique vasculature system, and focused exposure to light, this tissue complex, and in particular the central macular region, is predisposed to degeneration [2,3]. The age-related form of macular degeneration (AMD) is the leading cause of irreversible blindness in developed countries, and it is now estimated that 6.5% of the US population, aged 40 years and older, have AMD [4]. The most common AMD phenotype, generally termed 'dry AMD', is characterized by an increase in the number and diameter of extracellular sub-RPE deposits called drusen, pigmentary irregularities, progressive atrophy of the RPE and retina, and a graded loss in visual acuity [5-10]. In advanced cases, AMD is often associated with sub-retinal choroidal neovascularization (CNV; or 'wet AMD') and/or a clearly demarcated area of geographic atrophy (GA) in the macular region of the RPE. Both advanced AMD phenotypes cause severe vision loss.

Although aging is the prevailing risk factor for AMD, environmental factors such as smoking or oxidative stress may contribute to AMD's occurrence and/or progression [11-14]. Moreover, genetic linkage analysis and genome-wide association studies have identified a number of important genetic risk factors in recent years. The discovery of genetic variants in complement factor H, for example, firmly established a link between the complement cascade and AMD biology [15-18]. Other studies identified AMD risk variants in additional complement-related genes (for example, *C2*, *CFB*, *CFHR1*/*3*, *C3*) [19-22] as well as in a variety of non-complement-related genes, including a locus of unknown functional relevance (for example, *ARMS2*/*HTRA1*) [23-26] and loci related to lipid metabolism (*APOE*, *LIPC*, *ABCA1*) [27-33]. Despite these important discoveries, a detailed view of the biological pathways that mediate AMD development and progression has remained obscure. Furthermore, due to the morphological diversity of AMD clinical phenotypes, whether AMD represents a single disease consisting of multiple phenotypes or a disorder composed of distinct macular diseases (for example, dry AMD, CNV, and GA) is still unclear.

Compared to previous studies of AMD that have relied upon indirect experimental systems (for example, animal models, cell culture systems) and a reductionist experimental approach, gene expression profiling of human ocular tissues has great potential to more accurately and comprehensively resolve AMD-associated molecular signaling pathways. Coupled with a systems biology analysis, transcriptome profiling can be used for the unbiased identification of gene co-expression modules, to build molecular models with predictive utility, and to elucidate functional networks [34,35]. Although several groups have completed transcriptome-wide studies of relevance to AMD, including the identification of macular and extramacular differences in RPE-choroid gene expression, RPE-specific expression signatures, and AMD-associated changes in circulating leukocytes [36-40], no direct transcriptome-wide analysis of human RPE-choroid and retina AMD tissues has been reported to date.

Here we present our findings from a comparative transcriptome analysis of ocular tissues derived from 68 human donor eyes, including 26 well-characterized AMD eyes and 11 potential pre-AMD eyes. Our study identifies cell-mediated immune responses as the central feature of all AMD phenotypes, thus supporting the hypothesis that AMD is a single disease with a common immunological core process. In addition, in the RPE-choroid, we identified transcripts related to apoptosis and angiogenesis that are over-expressed in GA and wet AMD, respectively. In the retina, we observed a graded over-expression of wound response, complement, and neurogenesis genes that correlates with reduced levels of phototransduction transcripts and increasingly advanced AMD phenotypes. Finally, using these functionally enriched expression signatures, we assembled two detailed interactomes that highlight modular functional networks of classical dry AMD, CNV, and GA in RPE-choroid and neural retina tissues. These data provide new insights into the expression landscape of AMD pathophysiology, and reveal numerous new targets for the development of AMD-directed pharmaceuticals and diagnostics.

## Methods

### Donor eye tissue and RNA purification

RPE-choroid and retinal samples were isolated from human donor eyes obtained from the University of Iowa (GSH) and the Lions Eye Bank of Oregon. The Iowa eyes were selected from a well-characterized repository derived from over 3, 900 donors. Medical and ophthalmic histories, a family questionnaire, blood, and sera were obtained from the majority of donors. All Iowa donors were independently classified by two retinal specialists using gross pathologic features, and for 63 of the 68 Iowa donors, fundus photographs were utilized for grading purposes using well-established methods and morphological criteria [5,41,42]. The combined analysis of the retinal specialists was adjudicated (GSH) and donors were placed into groups based on the morphological phenotype of the eye with the most advanced pathology. Table 1 provides details of the grading scheme, and cross-references our groupings with the AREDS and Rotterdam grades, where visual impairment plays an additional role in classification. For each donor, only the eye with the most advanced phenotype (that is, the eye on which the classification was based) was used for transcriptome analysis. Macular trephine punches (8 mm) and temporally adjacent extramacular trephine punches (6 mm) of the RPE-choroid and retina were collected from Iowa eyes within 4 hours of postmortem, flash frozen in liquid N_2_, and stored at -80°C. Total DNA-free RNA was purified using a Qiagen RNeasy miniprep and on-column DNA digestion according to the methods of the manufacturer (Qiagen, Inc., Valencia, CA, USA). The RPE-choroid isolation procedures and RNA purification methods for material originating from the Lions Eye Bank of Oregon are described in Radeke *et al. *[36]. No retina samples were acquired from the Oregon eyes. Unlike Iowa eyes, postmortem times for Oregon eyes ranged up to 8.7 hours (90% > 4 hours), whole Oregon eyes were stored in RNA stabilization buffer (RNAlater, Ambion, Inc., Austin, TX, USA) at 4°C prior to sample collection, and off-column DNA digestion was used. In addition, unlike the Iowa eyes, which were expertly graded, the Oregon eyes received only a general classification of AMD based on medical histories confirmed by ophthalmological records. Oregon eyes with an absence of AMD clinical history were considered normal. Since Oregon eyes received a less rigorous AMD classification than Iowa eyes, the Oregon cohort was reserved for validation purposes only. Donor specific details (for example, age, gender, and AMD phenotype) can be accessed through the Gene Expression Omnibus [GEO:GSE29801].

**Table 1:** AMD classification scheme

AMD classification	Alternative name	**AREDS level **[42]	**Rotterdam grade **[41]	Description	Donors per class^a^
Normal		1	0a	No features of AMD	31
MD1	Pre-AMD	1	0b	Hard macular drusen (< 63 μm) only	7
MD2	Sub-clinical pre-AMD	2	1a	Soft, distinct macular drusen (> 63 μm)	4 (1a only)
			1b	Macular pigmentary irregularities without soft drusen	
Dry AMD	Dry AMD (non-GA)	3, 4b	2a	Soft, indistinct (> 125 μm) or reticular macular drusen	17
			2b	Soft distinct macular drusen (> 63 μm) with pigmentary changes	17
			3	Soft indistinct macular drusen with pigmentary changes	17
GA	Geographic atrophy	4a	4	Sharply demarcated area of apparent absence of the RPE (> 175 μm) involving central macular region	2
CNV	Wet AMD	4a	4	Sub-retinal choroidal neovascularization	4
GA/CNV		4a	4	Geographic atrophy with choroidal neovascularization	3

^a^Number of Iowa cohort donors per AMD classification group.

This study was reviewed and approved by the institutional review boards at St Louis University, the University of Iowa, the University of Utah, and the University of California, Santa Barbara and conforms to the tenets of the Declaration of Helsinki. Written informed consent was obtained from all participants or surviving relatives.

### Microarray hybridization, quantification, and normalization

Global transcriptome profiling was carried out using the Agilent Whole Human Genome 4 × 44 K *in situ *oligonucleotide array platform (G4112F, Agilent Technologies, Inc., Santa Clara, CA, USA) using the reagents and methods of the manufacturer, with the exception that 'spike-in' controls were not used. For the tissue samples, a two-color universal reference experimental design was employed where the dyes used to label experimental and reference samples were alternated with each sample. The universal reference was derived from a pool of donor eyes and consisted of a 50:50 mixture of RPE-choroid and retina RNA purified from tissue remaining after the macular and extramacular punches were removed. After Lowess correction, background subtraction, and normalization using the reference RNA, the net intensity was expressed as a percentage of the sum of all signals times 100, 000 (Percentage of total × 100, 000). Detailed DNA microarray methods and microarray data associated with this publication are available through the Gene Expression Omnibus [GEO:GSE29801].

### Identification of contaminating genes

To improve data quality and overall signal-to-noise ratio, RPE-choroid and retina gene expression datasets were filtered for the following putative contaminants: retina-enriched genes in the RPE-choroid, RPE-choroid-enriched genes in the retina, and gender-specific genes (for example, *XIST*). Differentially expressed genes were determined using an unpaired, two-sided Student's *t*-test with unequal variance, and the resulting *P*-values were adjusted by permuting class labels 1, 000 times with the Fisher-Yates method [43]. Moreover, a false discovery rate (also, q-value or *Q*) was determined for each gene probe using the method of Storey and Tibshirani [44]. Unfiltered RPE-choroid and retina microarray datasets were combined, quantile normalized [45], and log_2 _adjusted, and differentially expressed genes between RPE-choroid and retina with *Q *≤ 0.02, permuted *P *≤ 0.01, and fold change ≥ 1.5 were identified (Figure S1 in Additional file 1). Of 7, 029 RPE-choroid-enriched and 7, 736 retina-enriched gene probes meeting these statistical criteria, those with mean expression levels of < 100 (that is, approximately 6.64 in log_2 _space) in the opposing dataset were flagged as contaminants. Genes with gender-specific expression differences were identified using the combined RPE-choroid and retina dataset, and all genes with *Q *≤ 0.0001, permuted *P *≤ 0.001, and fold change ≥ 1.5 were flagged as gender-specific contaminants (21 male-enriched and 11 female-enriched gene probes).

### Combinatorial class comparisons for disease gene identification

Unfiltered RPE-choroid and retina datasets were quantile normalized separately and log_2 _transformed. Gene probes flagged as contaminants, or with minimal differential expression across all arrays (sample variance ≤ 5), were excluded from further analysis. Donor samples diagnosed as GA/CNV (*n *= 3) were combined with the pure GA (*n *= 2) and CNV (*n *= 4) samples to increase the *n *in these categories. In addition, samples collected from one 43-year-old individual diagnosed with AMD were excluded due to an atypically early disease onset. After data preprocessing, all AMD/pre-AMD phenotypes (pre-AMD (MD1), sub-clinical pre-AMD (MD2), MD1 + MD2 (MD), Dry AMD, GA, CNV), both separately and combined (global), were tested for significant differential expression against macular and/or extramacular age-matched normal donor samples (≥ 60 years), for a total of 21 two-class comparisons per gene probe per microarray dataset (RPE-choroid and retina). Statistical methods for differential expression analysis are described in 'Identification of contaminating genes'. A table of all gene probes with a permuted *P *< 0.1 and fold change ≥ 1.5 is provided as Table S1 in Additional file 2.

### Identification of AMD disease modules

Differentially expressed genes (listed in Table S1 in Additional file 2) were organized into a matrix consisting of the *P*-value for each gene (row *i*) and class comparison (column *j*). Each *P*-value was converted into a significance score *S_ij_*, calculated as -Log_10_(*P_ij_*), and all scores were assigned directionality based on the up- or down-regulation of each disease gene (positive or negative, respectively; non-significant genes have *S *= 0). Gene probes representing the same gene were collapsed by averaging significance scores, resulting in a matrix consisting of 42 columns (21 comparisons × 2 tissue types) and 6, 479 rows (unique genes/probes). The matrix was adjusted for AutoSOME clustering [46] using unit variance normalization (columns) and sum of squares = 1 normalization (rows and columns). All rows were subsequently clustered with AutoSOME using 500 ensemble runs, *P *< 0.005, and otherwise default parameters [46]. For each tissue type, clusters with genes primarily over- or under-expressed in the same phenotype were combined into larger groups termed 'disease modules'.

### Immunoglobulin gene probes

In one disease module (RPE-choroid Global Up), we observed a number of immunoglobulin-related gene probes along with many unannotated probes with highly similar expression profiles. Using AutoSOME [46], all gene probes with similar co-expression patterns to known IG probes were identified by clustering the expression data from the Global Up module (cluster parameters: *P *< 0.05, 500 ensemble runs, and otherwise default parameters). BLAT searches of the human genome reference sequence using the UCSC Genome Web Browser confirmed probe homology to *IGJ*, as well as immunoglobulin heavy, kappa, and lambda chain sequences. In total, 31 IG probes were found, and their expression values were averaged in three figures to conserve space. The 31 individual probes are highlighted in Figure S7 in Additional file 1.

### Disease state prediction

To validate candidate AMD biomarkers, we used the GenePattern implementation of support vector machine (SVM) [47], a machine learning algorithm for sample classification and prediction based on complex pattern recognition. Iowa expression data were log_2 _transformed and median-centered for the twenty most significant genes from the RPE-choroid Global Up module. Using known donor classifications (that is, Normal versus AMD/pre-AMD), the expression data, with and without age, were split into three training and test groups for stratified three-fold cross-validation (SplitDatasetTrainTest: split method = cross-validation; folds = 3; otherwise default parameters). SVM models were built for the training data (using GenePattern default parameters), and run on the corresponding test datasets, the full Oregon dataset (expression data processed identically to Iowa set), and a negative control consisting of randomized Oregon data (20 random genes with and without scrambled ages). Cross-validation accuracy was computed as the total number of correct classifications from all three Iowa test datasets divided by the number of Iowa RPE-choroid array samples (*n *= 126). SVM classification accuracy for the validation cohort (Oregon data) was calculated as the average accuracy obtained using the three SVM models. To calculate the statistical significance for overall classifier performance on each dataset, results from each of the three models were randomized by permuting class labels 10 million times, and a *P*-value was determined as the fraction of randomized results with a classification accuracy (averaged over the three models) equal to or exceeding the original non-randomized classification results [48]. *P*-values were also determined for individual SVM models using the same approach without averaging classification results. Finally, we note that our selection of the top 20 RPE-choroid Global Up genes for this analysis was not arbitrary, but was determined by iteratively running SVM (using cross-validation) on all consecutive subsets of RPE-choroid Global Up genes in the Iowa dataset, from the top singleton gene, to the top two genes, to the top three genes, and so forth. Using gene expression data alone, the highest classification accuracy was achieved using the top 20 and 21 genes (identical results; data not shown).

### Identification of disease modules enriched in protein-protein associations

Each disease module was analyzed for statistical enrichment of protein-protein associations using the STRING v8.3 database [49] (default parameters). Importantly, genes in GA&CNV Up/Down modules were not analyzed independently, but were included in GA and CNV Up/Down modules. Since STRING does not estimate statistical significance, as a proxy for enrichment, we used the average node degree *D*, defined as 2 × Number of edges/Number of nodes (edges = interactions; nodes = proteins). Monte Carlo sampling was employed to estimate the null distribution of *D *by sampling 50, 100, 200, 300, and 400 randomly drawn proteins 10, 000 times from the STRING database. Regression analysis revealed perfect linear trends for expected node degree *E*(*D*) for *P *= 0.05 (R^2 ^= 1; *E*(*D*) = 0.0023 × *nodes *+ 0.7318) and *P *= 0.01 (R^2 ^= 1; *E*(*D*) = 0.0022 × *nodes *+ 0.9068), and for each *P*-value we determined *E*(*D*) given the actual number of nodes within each disease module (that is, genes with matching proteins in STRING). Figure S11 in Additional file 1 illustrates the results of this analysis for the RPE-choroid (Figure S11a) and retina (Figure S11b), expressed as the deviation in node degree from random chance.

### Assembly of RPE-choroid and retina AMD interactomes

To construct high-quality AMD interactomes, we integrated both direct and indirect protein-protein interaction (PPI) data from three sources: Ingenuity Pathway Analyzer (IPA; Ingenuity Systems, Inc., Redwood City, CA, USA), STRING v8.3 [49], and a curated human PPI dataset [50]. Redundant protein-protein associations were eliminated in the final interactomes according to the following dominance series: Bossi and Lehner PPI > IPA > STRING database/experiment evidence > STRING text-mining evidence. Thus, Bossi and Lehner PPI data were given precedence over all other PPI data sources.

#### RPE-choroid interactome

Three RPE-choroid disease modules (Global Up, CNV Up, and GA Up) were found to have significant enrichment in protein-protein associations (*P *< 0.01; Figure S11 in Additional file 1). We note that CNV Up and GA Up modules include genes from the RPE-choroid GA&CNV Up module. Using at most 70 nodes and only interactions derived from *Homo sapiens*, an IPA Core Analysis was run for each of the three disease modules. For inclusion in the RPE-choroid interactome, we restricted IPA results to the highest scoring IPA network for a given Core Analysis. We also required a low ratio of predicted to observed proteins/complexes. Using these criteria, Global Up and CNV Up modules achieved high-quality networks. To reduce IPA network complexity and thus improve human readability, we eliminated self-edges as well as predicted proteins unnecessary for linking disease module gene products into the network. Additional interactions for each disease module were determined using STRING, with a minimum score of 0.7 ('high confidence' score). Since RPE-choroid GA Up is prominently enriched in apoptosis, all STRING interactions among RPE-choroid GA Up genes found in the 'apoptosis' Gene Ontology category (determined using ToppFun [51]) were added. Additional interactions among CNV Up proteins were also added by assembling a gene set consisting of CNV Up genes found in the IPA network along with additional CNV Up genes associated with the 'Wnt signaling pathway' and 'extracellular matrix' functional enrichment categories (determined with DAVID [52]). STRING interactions among CNV Up gene products derived from experimental or database evidence (score ≥ 0.7) were preferentially added; however, interactions derived from STRING text-mining (text score ≥ 0.7) were also added to incorporate one remaining protein, FGF9. Given the abundance of interactions identified by IPA for Global Up, no STRING interactions were added to the Global Up subnetwork. Finally, interactions among all three disease modules from the PPI dataset of Bossi and Lehner [50] were added. The final RPE-choroid interactome was rendered using Cytoscape 2.8.0 [53].

#### Retina interactome

Two retina disease modules, Global Up and CNV Up, were found to be significantly enriched in protein associations (*P *< 0.01; Figure S11 in Additional file 1). Given the enrichment of retina CNV Down in phototransduction processes and the critical importance of phototransduction to a perceptible disease phenotype, we also analyzed retina CNV Down genes for inclusion in the retina interactome. Notably, CNV Up and CNV Down modules include genes from the retina GA&CNV Up and Down modules, respectively. Using the same network quality criteria as used for the RPE-choroid, the IPA Core Analysis did not yield high quality networks for any of the three retina disease modules. Next, we used STRING to identify protein-protein associations. Given the large numbers of genes in retina CNV Up and Down modules, we first isolated subsets of functionally enriched genes from both modules using ToppFun [51]. Since CNV Up is predominantly enriched in neural-related processes, we isolated CNV Up genes found in the following Gene Ontology categories: neurogenesis, generation of neurons, neuron differentiation, and transmission of nerve impulse. CNV Down genes associated with vision were also isolated using the Gene Ontology categories: sensory perception of light stimulus, phototransduction, and response to light stimulus. We included all STRING interactions found within a combined gene set consisting of all Global Up genes, neurogenesis-related genes from CNV Up and vision-related genes from CNV Down (we employed the default STRING score of 0.4 since a score of 0.7 resulted in 30% fewer connected proteins). Finally, we added all human PPI data [50] determined for genes in the STRING-derived interactome. The final retina interactome was rendered with Cytoscape 2.8.0 [53].

### Compilation of AMD-associated genes

Through an extensive PubMed search and survey of recent review articles, we assembled a large list of genes or gene products with a confirmed or putative association with AMD resulting from genetic linkage, expression differences (RNA or protein) or localization to drusen in humans [15-33,40,54-110]. The gene list is provided in Table S2 in Additional file 3. Rather than requiring validation in two or more studies, only a single reference of association was required for inclusion in this list, to allow for a more comprehensive collection. Fisher's exact test was used to calculate the significance of overlap between the 283 AMD-associated genes (HLA super-locus excluded from count) in Table S2 in Additional file 3 and the RPE-choroid and retina interactomes (83 and 92 total differentially expressed network elements, respectively); only annotated genes from the Agilent 4 × 44 k chipset were considered for the total population count (*n *= 19, 542).

## Results

### AMD transcriptome profiling

To investigate gene expression programs that characterize AMD, we assembled global transcriptome profiles of macular and extramacular RPE-choroid and neural retina tissues from normal and AMD human donor eyes. Of two donor cohorts examined in this study, the Iowa cohort consisted of 31 normal eyes and 37 atypical eyes organized into the following graded phenotypes: macular hard drusen (MD1), distinct macular soft drusen 65 to 125 μm without pigmentary abnormalities (MD2), dry AMD without geographic atrophy (Dry AMD), wet AMD (CNV), and dry AMD with geographic atrophy (GA) (see Table 1 for detailed phenotype descriptions). The latter three classes correspond to clinically recognized AMD phenotypes while the former two classes, MD1 and MD2, represent potential pre- or sub-clinical stages of AMD. Although occasional hard drusen are common in the adult population, elevated numbers of hard drusen, and in particular macular hard drusen (that is, MD1), are not common and have been associated with an increased risk for developing AMD [111-114]. In addition, morphological features of MD2 are classically associated with AMD, but have not yet reached the size or extent necessary for a clinical diagnosis. These two phenotypes were therefore included in this study to explore potentially early events leading to the development of clinical AMD. Unlike Iowa eyes, donors from the second cohort were reserved for validation purposes (Oregon eyes, *n *= 30, RPE-choroid only). As a measure of quality control, we evaluated samples from both cohorts for postmortem RNA degradation, inter-array concordance, and agreement with previously published transcriptome experiments [36,38,115-117], and found our data to be of high quality and suitable for genomics analysis (see Additional file 4 for a detailed discussion of sample and array quality control measures).

**Figure 1:**
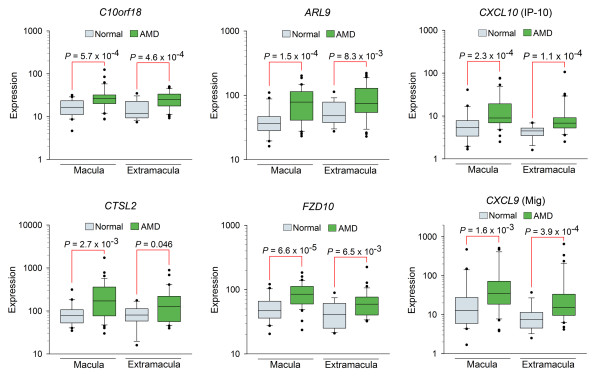
**RPE-choroid genes with significantly elevated transcript levels in AMD**. Box plots of age-matched normal and AMD (including pre-AMD) donor samples from the macular and extramacular RPE-choroid. All genes have a q-value ≤ 0.1 (false discovery rate ≤ 10%). Macular and extramacular *P*-values were calculated using a one-sided Wilcoxon rank-sum test. 'Expression' denotes net intensity, as defined in Materials and methods. Normal macula, *n *= 30; AMD macula, *n *= 35; Normal extramacula, *n *= 29; AMD extramacula, *n *= 32.

Employing an exhaustive series of pairwise class comparisons between age-matched normal donor samples (≥ 60 years) and the five graded AMD/pre-AMD phenotypes from the Iowa cohort, we identified numerous candidate AMD-associated genes, all of which satisfy a minimum fold change of 1.5 and permuted *P*-value cutoff of 0.1 (Table S1 in Additional file 2). Using a false discovery rate of 10% to account for multiple hypothesis testing (that is, *Q *≤ 0.1), we found seven genes in the RPE-choroid that exhibit differential expression across all AMD phenotypes, regardless of macular or extramacular origin. Six of these genes are expressed at higher levels in AMD (Figure 1), including two genes (*CXCL10 *and *CXCL9*) that encode angiostatic chemokines involved in leukocyte recruitment and implicated in diverse pathologies [118]. Additional genes found at higher levels include chromosome 10 open reading frame 18 (*C10orf18*), ADP-ribosylation factor-like 9 (*ARL9*; inferred from unannotated probe A_23_P58137, see Figure S5 in Additional file 1 for evidence), frizzled homolog 10 (*FZD10*), and cathepsin L2 (*CTSL2*). Only one gene was found consistently expressed at lower levels in the AMD RPE-choroid (gene probe A_24_P925565). We also found four globally differentially expressed genes between diseased and normal samples in the retina for *Q *≤ 0.1 (three genes of increased abundance, *LOC100294179*, *HLA-A*, and *ITGB1BP2*, and one gene with lesser abundance, *GSTT1*). Furthermore, using *Q *≤ 0.1, most of the gene expression changes associated with specific AMD phenotypes were found in the sub-clinical AMD state (that is, MD2), and for some disease phenotypes, no genes showed differential expression (for example, CNV in RPE-choroid).

### Identification of AMD disease modules

**Figure 2:**
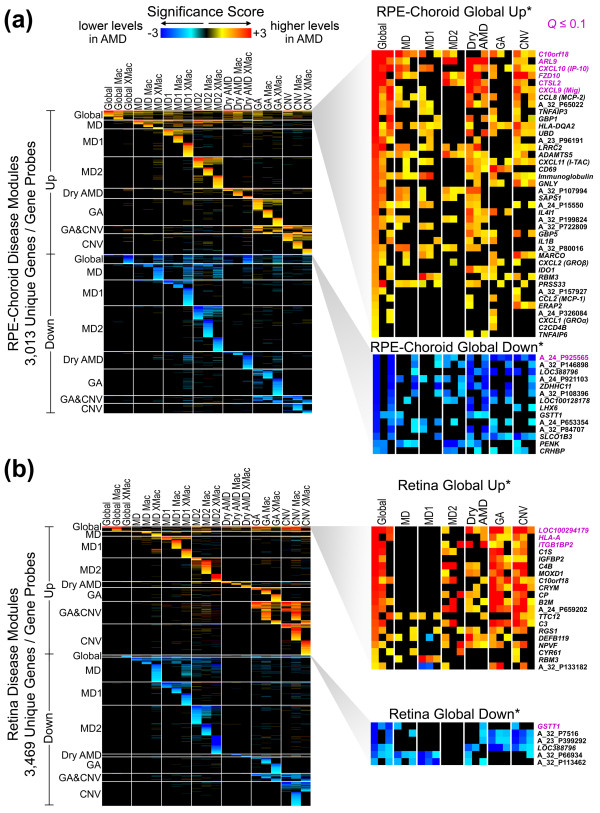
**Global and phenotype-specific AMD disease modules**. **(a, b) **Disease-associated genes with permuted *P*-value < 0.1 and fold difference ≥ 1.5 were clustered based on their significance score (see Materials and methods), and the results are displayed as heat maps for RPE-choroid (a) and retina (b). Columns represent each disease phenotype, macula (Mac) and/or extramacula (XMac), and rows represent unique genes/probes (gene symbols/Agilent ID). Global includes all AMD/pre-AMD phenotype classifications, and MD is composed of both MD1 and MD2 donor samples. GA and CNV disease classes include GA/CNV donor samples. Disease modules are separated by horizontal lines and labeled by higher or lower expression in one or more AMD/pre-AMD phenotypes (for example, 'MD2 Up', 'GA Down') compared to age-matched normal donor samples. Global Up*/Global Down* disease modules enlarged on the right highlight individual genes with significant differential expression in both macular and extramacular donor samples; for the entire list of Global Up/Down genes, see Figures S7 and S8 in Additional file 1 and Table S3 in Additional file 5. Immunoglobulin probes are averaged to conserve space, as described in Materials and methods.

Transcriptional heterogeneity in human donor eye tissue can arise from multiple factors, including normal genetic variation, environmental influences unrelated to AMD, the presence of mixed cell types, and/or variable degrees of AMD progression. Hence, the small number of candidate disease genes identified using *Q *≤ 0.1 was not unexpected. As an alternative approach, we relaxed the differential expression threshold for individual genes, and focused on identifying gene groups that exhibit both coordinated expression in specific disease phenotypes and significant functional enrichment in one or more biological processes. In particular, the q-value threshold was eliminated, and all differentially expressed genes identified in the class comparison with permuted *P *< 0.1 (Table S1 in Additional file 2) were clustered based on significance scores using AutoSOME [46]. Resulting clusters were then analyzed for functional enrichments. This approach, inspired by Segal *et al. *[119], allows for the determination of gene expression programs that may be obscured by noise. All clustering results are illustrated in Figure S6 in Additional file 1 and the corresponding gene lists and data are provided in Table S3 in Additional file 5. To highlight modularity, clusters with genes differentially expressed in the same disease phenotype(s) were further combined into AMD disease modules, for both RPE-choroid (Figure 2a, left) and retina (Figure 2b, left).

### Elevated cellular immune response is associated with all disease phenotypes

Although most of the 32 identified disease modules are restricted to discrete AMD phenotypes (Figure 2, left), four global disease modules were identified that consist of genes differentially expressed in multiple AMD and pre-AMD phenotypes (Figure 2a, b, right panels; also see Figures S7 and S8 in Additional file 1). Among the more than 50 array probes corresponding to annotated genes in the RPE-choroid Global Up module, Gene Ontology analysis revealed a striking enrichment in genes regulating cell-mediated immune processes (for example, chemokine activity, *P *= 3.0 × 10^-11^; Table S4 in Additional file 6). This module includes genes for all known CXCR3 ligands (the previously identified *CXCL9 *and *CXCL10 *shown in Figure 1, along with *CXCL11*); *CCL2*, which encodes a pro-inflammatory chemokine previously associated with AMD [64]; *CD1D *and *CD86*, which are both associated with antigen presenting cells [6,120,121]; and immunoglobulins (*IGJ*, *IGH@*, *IGK@*, *IGL@*). Similarly, the Retina Global Up module is significantly enriched in inflammatory genes (*P *= 1.3 × 10^-4^). However, unlike the RPE-choroid Global Up module, these genes are involved in the complement and coagulation cascades (*C3*, *C4B*, *C1S*, *CFI*, *F5*, *SERPINA5*; Table S4 in Additional file 6). In contrast to Global Up genes, Global Down genes are not functionally enriched, aside from three neurofilament-associated genes in the retina (*NEFL*, *NEFM*, *PRPH*), suggesting that AMD is associated with a general down-regulation of genes involved in diverse cellular processes. Notably, one gene present in both RPE-choroid and Retina Global Down modules, *GSTT1 *(glutathione S-transferase theta 1), is known to play a protective role in RPE oxidative stress, and reduced levels of this gene may be associated with both advanced RPE aging [122] and AMD [85]. In summary, by examining clusters of co-expressed genes for functional enrichment, these results greatly expand upon the global disease genes previously identified using *Q *≤ 0.1 (Figure 1), and indicate that there are multiple biomarkers of AMD. Furthermore, these data are consistent with a global cellular immune response in AMD pathogenesis.

### Validation of RPE-choroid global signature genes using an independent cohort

**Figure 3:**
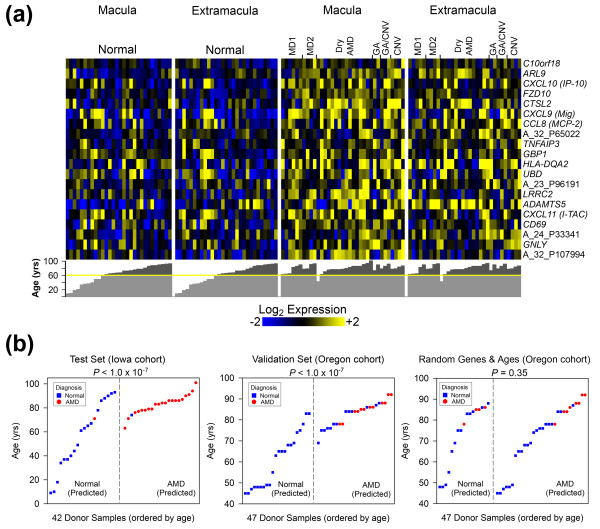
**Validation of global AMD signature genes with an independent cohort**. Using a support vector machine (SVM) and the 20 most significant genes in the RPE-choroid Global Up module (Figure 2a), a classification model for predicting AMD status was developed. **(a) **Expression heat map (log_2 _scaled, median-centered) of the genes/probes from the Iowa donor set used to generate the classification model. All donor ages are plotted under the heat map (yellow line indicates point at which donors become age-matched). **(b) **Results obtained using an AMD SVM model that incorporates expression data and age to identify AMD donors in: a subset of the Iowa data that was not used for training (that is, 'Test Set'), the Oregon dataset (that is, 'Validation Set'), and a randomized Oregon dataset (that is, 'Random Genes and Ages'). These results correspond to 'SVM Model 2 (+Age)', which is detailed along with other SVM models in Figures S9 and S10 in Additional file 1. Statistical significance was determined as described in Materials and methods.

To validate our finding of a global AMD expression signature, we used a SVM [47] classifier applied to the macular and extramacular expression profiles from the top 20 genes in the RPE-choroid Global Up disease module (Figure 3a). In addition to the set of donor samples used in our initial transcriptome analysis (Iowa set), we analyzed a validation cohort of RPE-choroid samples collected from both normal eyes and eyes with a clinical diagnosis of AMD obtained from the Lions Eye Bank of Oregon. Using stratified three-fold cross-validation, we trained SVM models on three different subsets of Iowa RPE-choroid samples, and evaluated each model on the remaining Iowa samples not used for training (one unique test set for each model, *n *= 42), the full Oregon dataset (*n *= 47), and a negative control Oregon dataset (*n *= 47) composed of 20 randomly selected genes. Moreover, since age is the most prominent risk factor for AMD, we also tested SVM performance with the inclusion of donor age as a covariate.

All SVM models achieved statistically significant AMD discrimination on every dataset except the randomized negative control (Figures S9 and S10 in Additional file 1). Based on the percentage of AMD donor samples that were correctly identified, SVM models generated from expression data alone achieved a cross-validation classification accuracy of 81% on the Iowa test data (*P *= 1.6 × 10^-5^; Figure S9 in Additional file 1), an average classification accuracy of 71% on the Oregon dataset (*P *= 6.3 × 10^-4^; Figure S10a in Additional file 1), and an average accuracy of 55% for 20 randomly selected Oregon genes (*P *= 0.24; Figure S10b in Additional file 1). After incorporating age, the cross-validation accuracy of the Iowa test data improved to 84% (*P *= 3.3 × 10^-7^) and the average accuracy of the Oregon data improved to 72% (*P *= 3.6 × 10^-4^). It is worth noting that the predictive accuracy of our SVM models may be underestimated on the Oregon cohort due to possible non-diagnosis of AMD. For example, many of the RPE-choroid Global Up disease module transcripts are also found at elevated levels in what might be pre- or sub-clinical stages of AMD (for example, MD1 and MD2; Figures 2a and 3a). Since most misclassified Oregon donors represent the oldest individuals at highest risk for developing AMD (Figure 3b; Figure S10a in Additional file 1), and because Oregon donors were not graded postmortem, such individuals may have exhibited early stages of AMD. Regardless, these data collectively validate our approach for the identification of AMD-related genes, and demonstrate that genes within the RPE-choroid Global Up disease module are global biomarkers of AMD.

### Functional enrichments of AMD phenotype-specific modules

In addition to Global Up modules, nearly three-quarters of the remaining disease modules (19/26) have significant functional enrichments (Table S4 in Additional file 6). Like the RPE-choroid Global Up module, inflammation is a prevalent functional category for other AMD phenotype-specific RPE-choroid modules (pre-AMD (MD1), Dry AMD, GA, and CNV Up), indicating that distinct inflammatory elements may contribute to AMD phenotypic diversity.

In particular, the two advanced AMD phenotypes, GA and CNV, are highly enriched in functional pathways and cellular activities. The RPE-choroid CNV Up module contains numerous genes associated with vascularization, including genes associated with extracellular matrix (*P *= 4.6 × 10^-7^) and circulatory system development (*P *= 2.5 × 10^-5^). The RPE-choroid GA Up module is robustly enriched in apoptotic processes (*P *= 9.6 × 10^-9^), consistent with previous observations implicating cell death in geographic atrophy [79,123]. In retinal tissues, both CNV and GA show elevated expression of major histocompatibility complex I genes (*P *< 0.01) and genes involved in neurogenesis (*P *< 0.05), along with a concomitant decrease in transcripts critical for photoreceptor function in the macula (GA, *P *= 1.5 × 10^-4^; CNV, *P *= 7.3 × 10^-9^), including red and green cone opsins (*OPN1LW *and *OPN1MW*) previously associated with the degenerative effects of drusen [124]. The Retina CNV Down module also includes rhodopsin (*RHO*) and photoreceptor outer segment genes. Importantly, no evidence for elevated photoreceptor transcript levels was seen in the corresponding RPE-choroid samples, ruling out loss of photoreceptors during dissection due to the presence of a disciform scar. These data thus reaffirm the characteristic phenotypes of these two advanced AMD phenotypes, and identify numerous differentially expressed genes for further study.

In contrast to the advanced AMD phenotypes, CNV and GA, our analysis did not reveal notable functional features of Dry AMD (non-GA) in the RPE-choroid beyond cell-mediated immune responses (Table S4 in Additional file 6). Similarly, prominent phenotype-specific functional enrichments were not observed in the retina, aside from a reduction of neuronal processes in both MD2 and pooled samples of MD1 and MD2 (MD Down). These results may reflect the heterogeneity of classification systems applied to delineate putative stages (or phenotypes) of MD1, MD2, and Dry AMD (Table 1).

### Functional networks delineate global and advanced-stage AMD phenotypes

**Figure 4:**
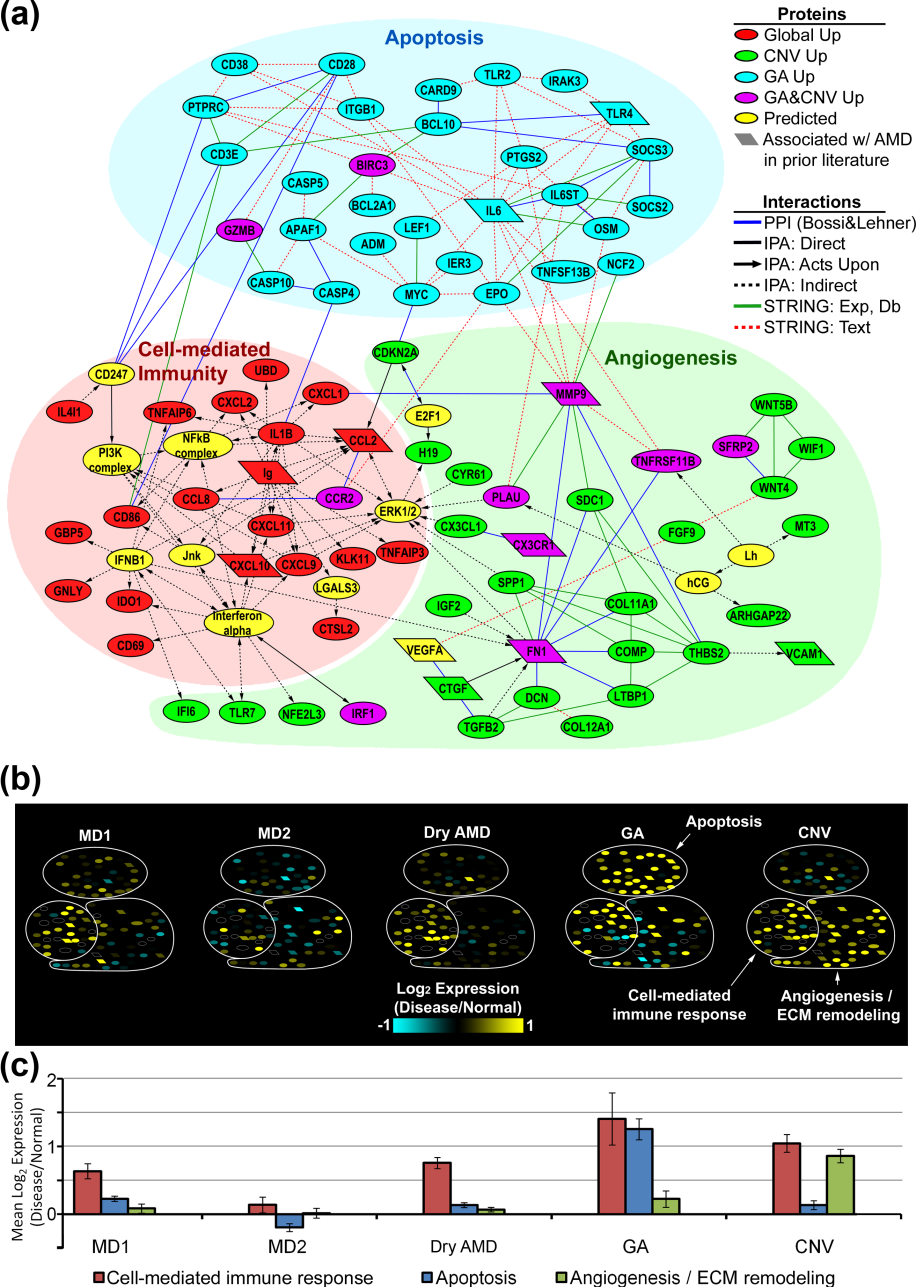
**RPE-choroid AMD interactome: globally conserved and phenotype-specific subnetworks**. **(a) **AMD interactome showing direct and indirect protein-protein interactions within RPE-choroid Global Up, CNV Up and GA Up disease modules assembled from three data sources: STRING [49], Ingenuity Pathway Analyzer (IPA), and the Bossi and Lehner [50] human protein-protein interaction dataset (PPI) (see Materials and methods). Gene products are represented by nodes, most of which are color-coded according to disease module. Yellow nodes represent genes predicted by IPA, with the exception of *VEGFA*, which was predicted manually and is highly expressed in our RPE-choroid expression data. Parallelogram-shaped nodes denote genes previously associated with AMD (Table S2 in Additional file 3). Individual immunoglobulin genes/probes in the Global Up module were combined (see Materials and methods) and are represented as a single node ('Ig'). Exp, Db, and Text indicate Experimental, Knowledge/Database, and Text-mining components of the STRING interaction score, respectively. **(b) **Heat maps depict differential expression of network genes in the macula of each AMD/pre-AMD phenotype (also see Figure S12 in Additional file 1). Differential expression was calculated as the geometric mean of each gene normalized to age-matched normal donor samples (≥ 60 years). Only pure GA and CNV are represented. (For data including GA/CNV donor samples, see Figure S12 in Additional file 1). **(c) **Bar plot depicting mean of expression data in (b), shown as a function of AMD/pre-AMD phenotype. Error bars represent standard error of the mean.

As a first step toward developing a systems-level molecular model of AMD, we examined the disease modules for evidence of protein-protein associations. We found six disease modules with significant enrichment in known and predicted associations (*P *< 0.01; Figure S11 in Additional file 1). Further analysis of the three most significant RPE-choroid modules (RPE-choroid Global, CNV, and GA Up) revealed a protein interactome consisting of both direct and indirect interactions among 95 proteins (83 disease module genes) divided into cell-mediated immunity, angiogenesis/extracellular matrix remodeling, and apoptosis subnetworks (Figure 4a). To explore gene expression within this interactome across each AMD and potential pre-AMD phenotype, normalized expression levels from the macular RPE-choroid were superimposed onto the network (Figure 4b) and mean levels were plotted (Figure 4c). (Both macular and extramacular expression levels, depicted as heat maps, are shown in Figure S12 in Additional file 1.) As illustrated in Figure 4b, c, transcripts in the cell-mediated immunity subnetwork are found at higher levels in the macula for every phenotype, except MD2 (consistent with Figure 2a, right). Furthermore, apoptosis and extracellular matrix remodeling subnetwork transcripts are more abundant in GA and CNV, respectively, consistent with our previous results (Table S4 in Additional file 6). Moreover, this interactome is strikingly enriched in genes associated with AMD in prior literature (10/83 differential expressed network elements; *P *= 3.3 × 10^-7^). A table of previously reported genes/gene products cross-referenced with the interactome and disease module genes is provided as Table S2 in Additional file 3 (see Materials and methods for details).

**Figure 5:**
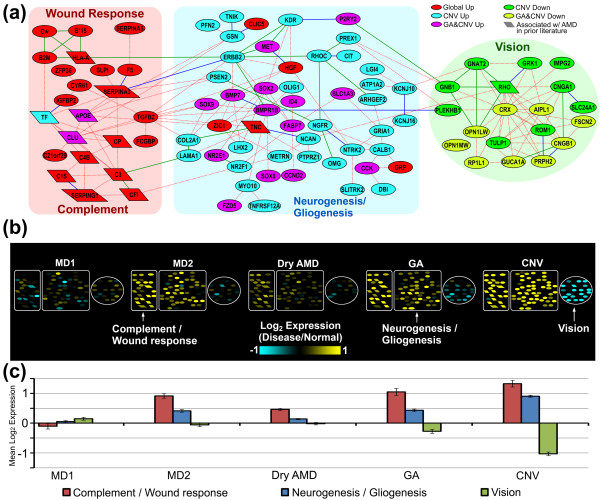
**Retina AMD interactome shows graded expression across AMD phenotypes**. **(a) **AMD interactome assembled from retina Global Up, CNV Up and CNV Down disease modules assembled from three data sources: STRING [49], Ingenuity Pathway Analyzer (IPA), and the Bossi and Lehner [50] human protein-protein interaction dataset (PPI) (see Materials and methods). Parallelogram-shaped nodes indicate genes previously associated with AMD (Table S2 in Additional file 3). Exp, Db, and Text indicate Experimental, Knowledge/Database, and Text-mining components of the STRING interaction score, respectively. **(b) **Heat maps depicting macular expression levels as a function of AMD and pre-AMD phenotype (also see Figure S13 in Additional file 1). Expression levels were calculated as described in Figure 4. **(c) **Mean differential expression for each subnetwork in (b), organized by AMD/pre-AMD phenotype. Error bars represent standard error of the mean.

Separately, we assembled a retina interactome (Figure 5a) consisting of significantly connected proteins from the Global Up and CNV Up modules (*P *< 0.01; Figure S11 in Additional file 1), along with phototransduction proteins functionally enriched in the CNV Down module. Normalized macular expression levels indicate a tight co-regulation of complement/wound response and neurogenesis subnetworks (Figure 5b, c; see Figure S13 in Additional file 1 for both macula and extramacula). Furthermore, there is a progressive decrease of the phototransduction subnetwork RNAs from Dry AMD to GA to CNV that is virtually macula-specific (Figure S13 in Additional file 1). Finally, like the RPE-choroid, the retina interactome significantly overlaps with previously studied AMD genes/gene products (13/92 network elements; *P *= 7.5 × 10^-10^; Table S2 in Additional file 3). These findings further validate our transcriptome data and support the conventional classification of AMD into functionally distinct and increasingly severe phenotypes.

## Discussion

In this study, we analyzed transcriptome profiles of the tissues most affected in AMD, the RPE-choroid and neural retina. Using pairwise statistical analysis in combination with significance score-based clustering, we identified 32 novel gene expression signatures, termed disease modules, associated with major clinical AMD phenotypes as well as two potential pre-AMD phenotypes (Figure 2). Among the 32 disease modules, we discovered 4 disease modules common to multiple disease phenotypes (Figure 2). Next, we showed by SVM analysis that expression levels of the top 20 genes in the RPE-choroid Global Up module (Iowa cohort) can significantly discriminate between clinically documented cases of AMD and normal donors in an independent RPE-choroid dataset (Oregon cohort). Finally, using disease module genes with statistically enriched functional concordance, we assembled detailed interactomes that reveal both global and phenotype-specific processes associated with AMD (Figures 4 and 5). Collectively composed of nearly 200 differentially expressed genes, these interactomes are statistically enriched in genes previously associated with AMD (23 total genes; Figures 4 and 5; Table S2 in Additional file 3), thus validating our analytical strategy and further implicating the previously unexplored network components in AMD pathology.

Our findings reveal that cell-based inflammatory responses within the RPE-choroid are a core feature of AMD. Remarkably, this global process is even detectable in one of the earliest potential stages of the disease prior to perceptible vision loss (MD1; Figures 4b and 5b). Thus, Oregon donors classified by SVM models as *AMD *(Figure S10a in Additional file 1), but initially labeled 'normal' (Materials and methods), may have been in the beginning stages of AMD. Recently, elevated levels of CXCL10 were reported in the sera and choroid of individuals with AMD [63], and elevated intraocular CCL2 levels were observed in neovascular AMD [64]. Here we show that all AMD phenotypes in the RPE-choroid are associated with elevated expression of all, or a subset, of the following chemokines: CXCL1, CXCL2, CXCL9, CXCL10, CXCL11, CCL2, and CCL8 (Figure 2a, right). These chemokines are known to recruit macrophages, dendritic cells, granulocytes, CD4+ Th1 cells, CD8+ T cells, and natural killer cells to damaged tissue [125]. Although activated macrophages and other leukocytes are known expressers, most of these chemokines are also expressed in cultures of human fetal RPE following exposure to IL1B [126]. (Notably, *IL1B *is found in the RPE-choroid Global Up module.) Determining the cellular source(s), context-specific cellular target(s), and regulatory mechanism(s) of AMD-associated chemokines remains an important goal of future work.

A number of other factors in the RPE-choroid Global Up module further implicate a cellular immune response in AMD. For example, *CD86 *is expressed in drusen-associated dendritic cells [6], *CD69 *is expressed in activated leukocytes [127], and *ILI41 *and the elastolytic protease *CTSL2 *are both expressed by macrophages upon activation [128]. In addition, the up-regulation of immunoglobulin genes supports an adaptive, autoimmune response in AMD that is consistent with previous reports of immunoglobulins in drusen and drusen-associated RPE [129] as well as anti-carboxyethylpyrrole adduct antibodies [130] and anti-retinal antigen auto-antibodies [131-133] in AMD sera.

We also identified a Retina Global Up module composed of more than 50 genes common to sub-clinical AMD (MD2), Dry AMD (non-GA), neovascular AMD, and GA, but not to donors at risk for AMD with macular hard drusen alone (MD1) with little to no vision loss (Figure 2b, right, and Figure 5b). In addition to wound response genes (for example, *TGFB2*, *CYR61*), this module is highly enriched in complement genes previously associated with AMD (*C3*, *C4*, *C1S*, *CFI*, *SERPING1*; Table S4 in Additional file 6). Given the prevailing view that the role of complement in AMD is linked to deposition of the terminal complement complex in drusen and the capillary pillars of the choroid [6-8,10], this finding is unanticipated, and indicates a possible direct role for complement in retinal degeneration. In addition, the up-regulation of both complement and major histocompatibility complex I genes (*HLA-A*/*B*/*C *genes, and *B2M*) in the Global Up module, along with *APOE *elevation in the Retina GA Up and CNV Up modules (Figure 5b) may reflect activation of resident microglia in the retina [134,135]. This is further supported by reports of a similar microglial immunological response in a *CX3CR1*^-/- ^mouse model of AMD [135], as well as in animal models of photo-induced retinal damage [136,137].

Finally, by integrating functionally enriched gene sets with interactome data, this work reveals many attractive candidates for AMD therapeutics and diagnostics (Figures 4 and 5). Examples of wet AMD candidate targets within the RPE-choroid interactome (Figure 4) include *IGF2*, an imprinted gene adjacent to *H19 *(also in Figure 4) whose product up-regulates *VEGF *expression [138], CYR61, a matricellular protein that modulates angiogenesis and apoptosis [139], and SPP1, a matricellular CFH binding protein that is both a promoter of VEGF-induced endothelial migration and an immunomodulator [140,141]. GA Up genes with potential relevance for pharmaceutical intervention include *EPO*, which inhibits oxidative damage-induced apoptosis in cultured RPE [142] and *IL6*, a mediator of RPE degeneration [143]. Furthermore, the interactome genes constitute only a small subset of all identified disease module genes with potential utility for AMD therapy. For example, 62 angiogenesis-related genes in the RPE-choroid CNV Up module encode secreted proteins (Table S3 in Additional file 5), any one of which may represent an effective drug target.

## Conclusions

In this study, we discovered novel global biomarkers, phenotype-specific gene sets, and functional networks associated with AMD. Although further studies will be needed to elucidate the specific cell populations responsible for gene expression changes in AMD, and to further validate and confirm the identity of these AMD-associated genes, our results represent a major step toward assembling a systems-level model of AMD, and establish a benchmark for future studies that incorporate both greater cohort sampling and higher-resolution genomic profiling (for example, employing microdissection and next-generation sequencing methodologies). Fundamentally, this work demonstrates that immune responsiveness is a central, unifying process that characterizes the molecular pathology of all AMD phenotypes [6,7,10], consistent with the hypothesis that AMD is a singular disease with multiple outcomes. In summary, these data support the model that aging, environmental stressors, and genetic predisposition all hasten the onset and progression of cell-based immunological events, leading to a state of chronic local inflammation that mediates the development of the neovascular and/or atrophic changes characteristic of advanced AMD. In light of these results, we suggest that pharmaceuticals targeting core immunological processes may have broad efficacy for all clinical manifestations of AMD.

## Abbreviations

AMD: age-related macular degeneration; CNV: choroidal neovascularization or 'wet AMD'; GA: geographic atrophy; IPA: Ingenuity Pathway Analyzer; MD1: pre-AMD; MD2: sub-clinical pre-AMD; PPI: protein-protein interaction; RPE: retinal pigmented epithelium; SVM: support vector machine.

## Competing interests

GSH received funding from Alcon Research Institute and Allergan, Inc. No other authors have any competing interests.

## Authors' contributions

AMN designed the study, analyzed the data and drafted the manuscript. NBG, LH, NM, CMR, and MAM assisted in laboratory research. JBC contributed to data interpretation and manuscript revision. GSH, DHA, and LVJ conceived of the study and contributed to manuscript revision. MJR conceived of and designed the study, analyzed the data and drafted the manuscript. All authors read and approved the final manuscript.

## Supplementary Material

Additional file 1 **Figures S1 to S13**. Supplemental figures and corresponding legends. Click here for file

Additional file 2 **Table S1**. Differentially expressed genes between AMD and age-matched normal donor samples in the RPE-choroid (sheet 1, Table S1, RPE-choroid) and retina (sheet 2, Table S1, Retina). Click here for file

Additional file 3 **Table S2**. Curated list of genes previously associated with AMD. Click here for file

Additional file 4 **Assessment of RNA and microarray quality**. Description of analytical procedures used to determine the quality of the RNA and microarray data. Click here for file

Additional file 5 **Table S3**. RPE-choroid (sheet 1, Table S3, RPE-choroid) and retina (sheet 2, Table S3, Retina) disease module gene lists and associated data. Click here for file

Additional file 6 **Table S4**. Disease module functional enrichments. Click here for file
