# Supplementary material for: Systems-level analysis of age-related macular degeneration reveals global biomarkers and phenotype-specific functional networks
Source: Genome Med. 2012 Feb 24;4(2):16. doi: 10.1186/gm315 (PMC3372225; doi:10.1186/gm315)
Supplement: Additional file 1 — Figures S1 to S13. Supplemental figures and corresponding legends. [file gm315-S1.DOC]

**Systems-level analysis of age-related macular degeneration reveals global biomarkers and phenotype-specific functional networks**

Aaron M. Newman, Natasha B. Gallo, Lisa S. Hancox, Norma J. Miller, Carolyn M. Radeke, Michelle A. Maloney, James B. Cooper, Gregory S. Hageman, Don H. Anderson, Lincoln V. Johnson & Monte J. Radeke

**Supplemental Figures S1 - S13**

**
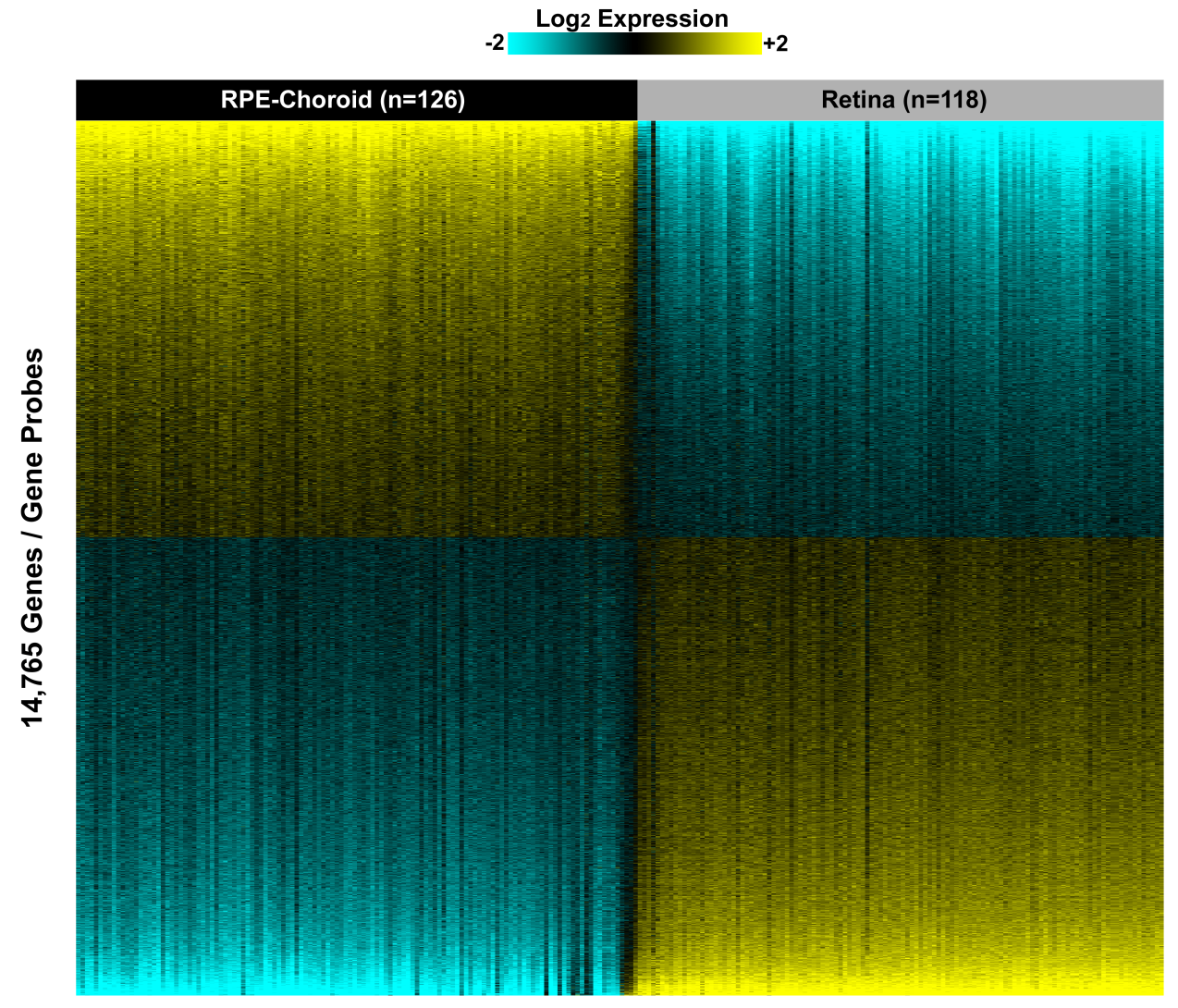
**

**Figure S1.** Heat map of significant differentially expressed genes between RPE-choroid and retina from the Iowa cohort. To identify and remove contaminating genes from adjacent retinal tissues, we identified significant gene expression differences between RPE-choroid and neural retina, followed by elimination of putative contaminants (see Methods). Differential expression was determined using Student’s t-test with unequal variance, followed by multiple hypothesis correction using class label permutations (1000x) and False Discovery Rate [43, 44]. All gene expression patterns depicted in the heat map meet the following statistical criteria: permuted p-value ≤ 0.01, q-value ≤ 0.02, and fold change ≥ 1.5. Macular and extramacular samples are included. For additional details, see *Materials and Methods*.

**
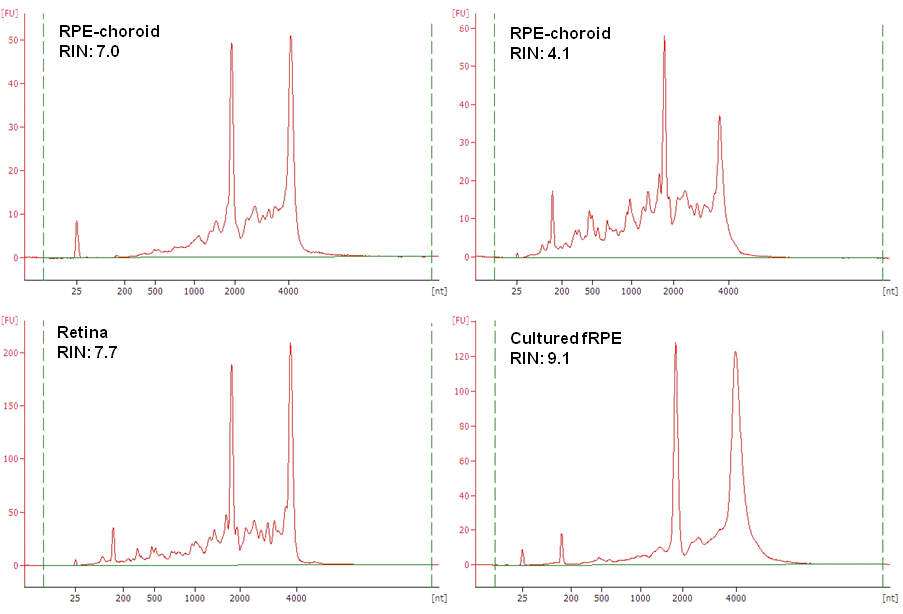
**

**Figure S2.** Quality control analysis of RPE-choroid and retina RNA. At least one RNA sample from 94 of the 96 donor eyes was assessed by microchannel electrophoresis. RNA index numbers (RIN) ranged from 4.0 to 8.7. Representative electropherograms, including that from the lowest quality RPE-choroid sample, are shown. An electropherogram obtained for RPE purified from culture fetal RPE is shown for comparative purposes. Although there is evidence of RNA degradation in all RNA samples derived from postmortem sources, intact ribosomal RNAs are still detectable in the lowest quality RNAs.


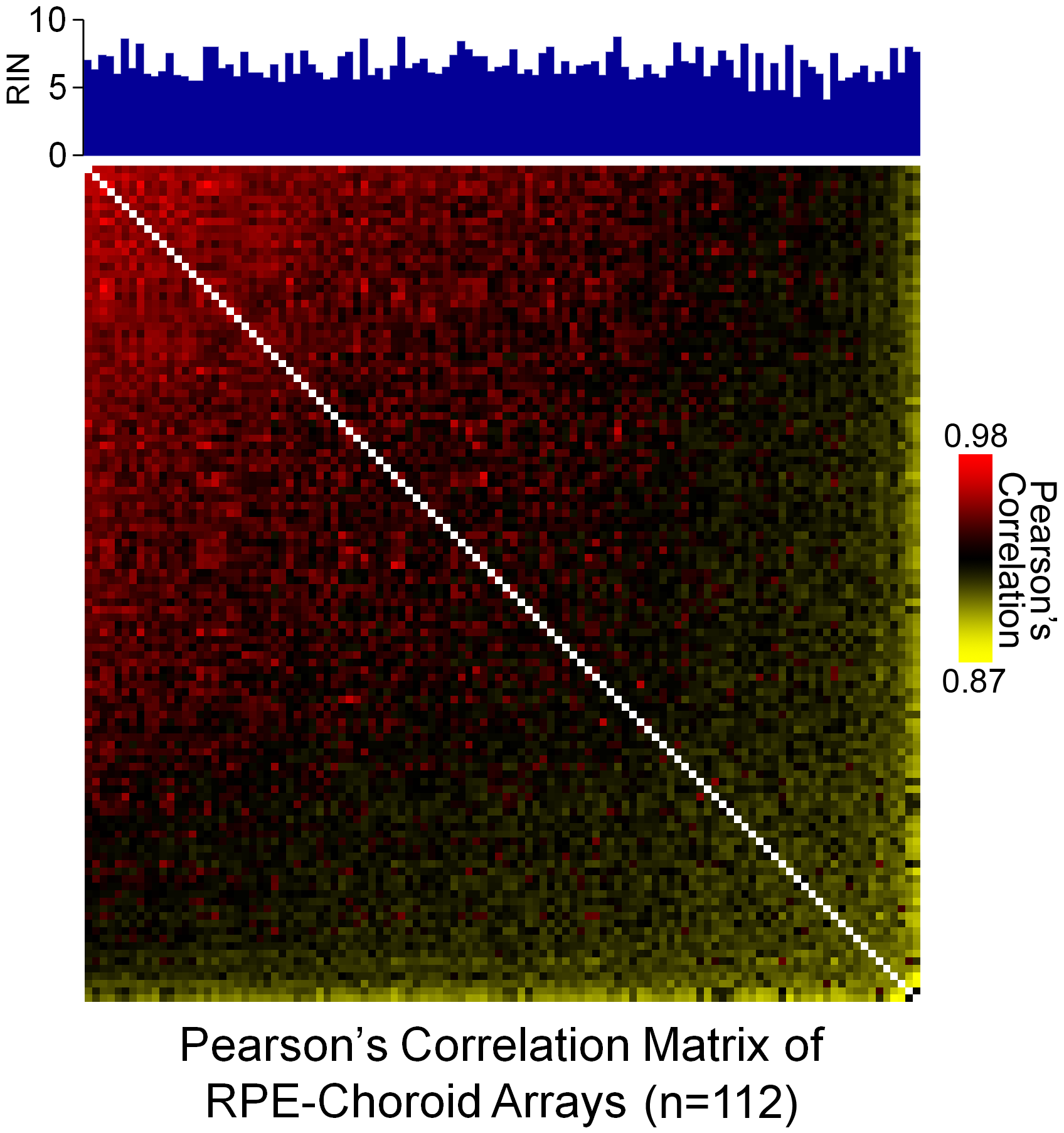


**Figure S3.** Inter-array differences, as determined by Pearson’s correlation, are not correlated with RNA Integrity Number (RIN). Heat map depicting the Pearson’s correlation for every pair of RPE-choroid arrays with a measured RIN (n = 112), arranged as a matrix with 112 rows and 112 columns. Corresponding RINs are plotted above the heat map. Higher correlation coefficients are shown in red, lower correlations are yellow, and self-comparisons are indicated in white along the diagonal. The heat map matrix is ordered from top to bottom and left to right by decreasing mean correlation, determined for each row and column, respectively. Following quantile-normalization and log2 adjustment, all 41,000 gene probes were used for calculation of correlation coefficients.


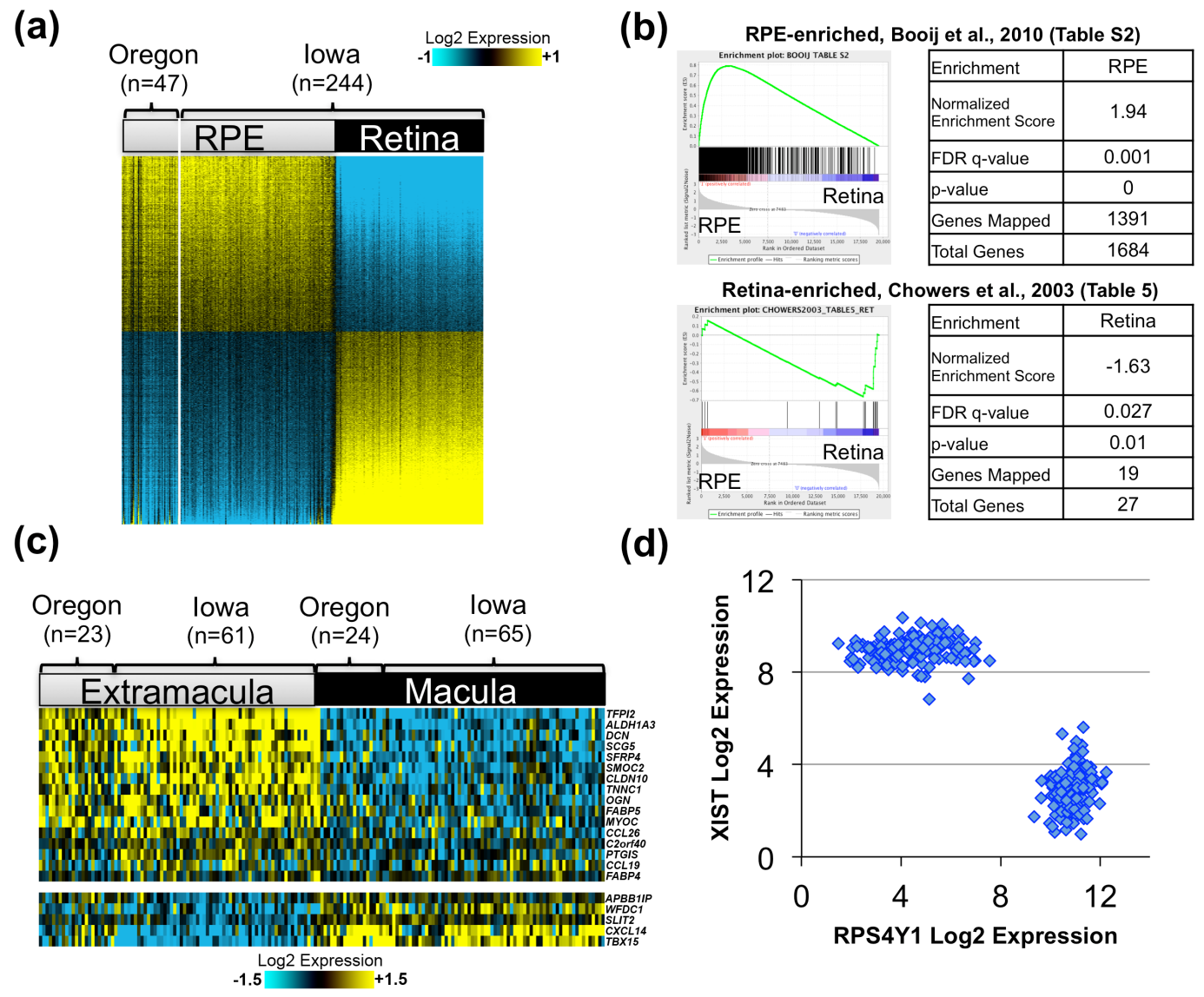


**Figure S4.** Microarray quality control analysis of RPE-choroid and retina. (a) Inter-array concordance, as illustrated by a heat map of differentially expressed genes between RPE-choroid and retina. The same genes shown for the Iowa data in Figure S1 are displayed here, along with the addition of Oregon RPE-choroid expression data for these genes. (b) Gene-set enrichment analysis (GSEA) comparing our microarray data to RPE and retina-enriched genes sets respectively published by Booij et al. [38] (upper sub-panel) and Chowers et al. [116] (lower sub-panel). A combined microarray dataset consisting of all 291 arrays (Iowa and Oregon cohorts, RPE-choroid and retina) was used for this analysis. (c) Genes that were previously validated by qPCR to exhibit differential expression between RPE-choroid macula and extramacula [36] are shown using our RPE-choroid microarray data and a heat map display. (d) Expression plot of *XIST* and *RS4Y1* for all 291 arrays. For all quality control exercises (panels a-d), corresponding microarray data were combined, quantile-normalized, and log2 normalized prior to analysis.

**
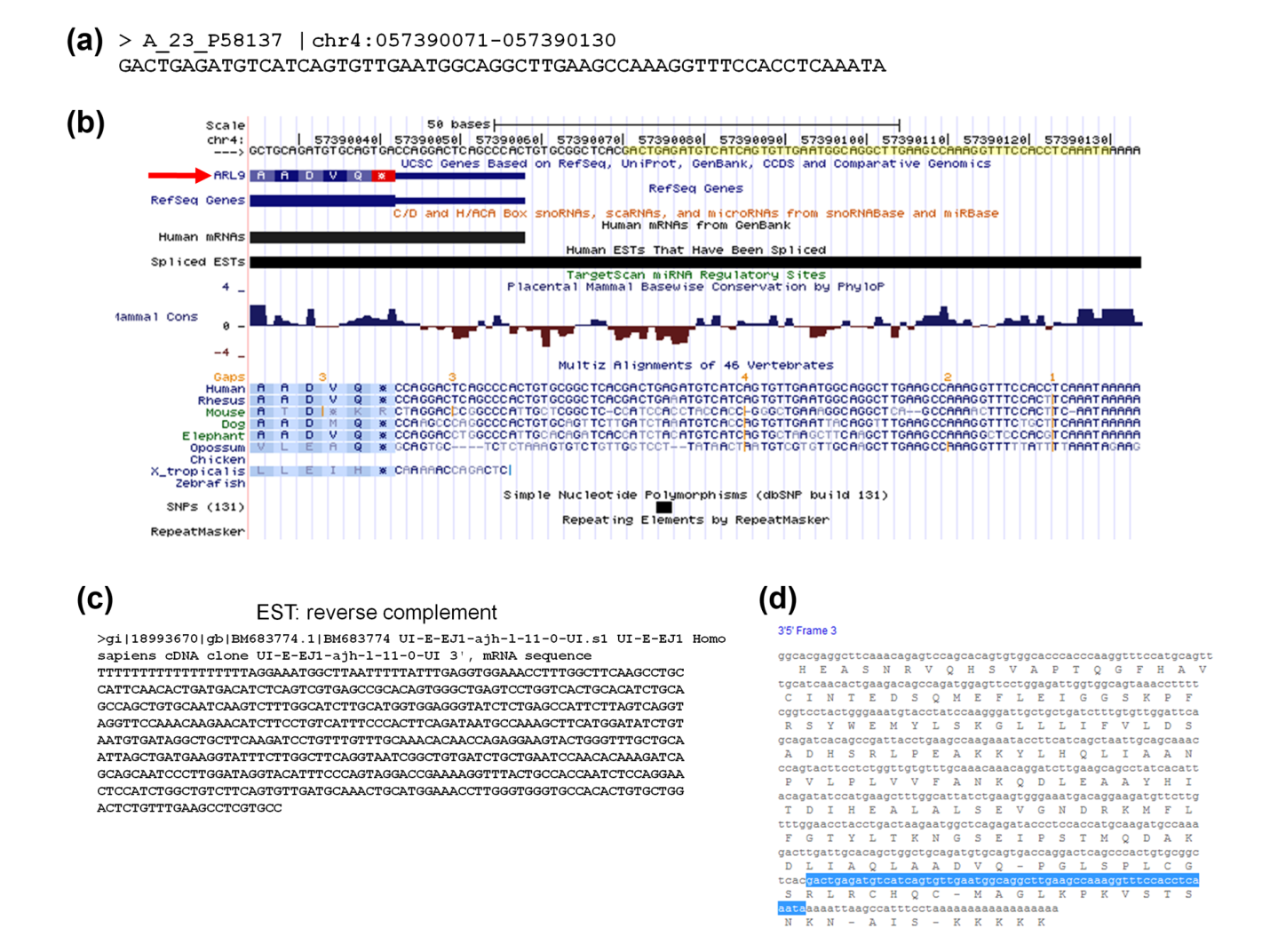
**

**Figure S5.** Evidence for ADP-ribosylation factor-like 9 (ARL9). (a) Unannotated Agilent gene probe A_23_P58137. (b) A_23_P58137 sequence (highlighted in yellow) maps downstream of the *ARL9* transcription termination site (UCSC genome browser). (c) A_23_P58137 is completely contained within the EST sequence gi:18993670, identified by BLAST analysis. (d) 3’5’ Frame 3 translation of gi:18993670 contains the genomic sequence shown in panel *B* (probe sequence highlighted in blue). The 3’5’ orientation of gi:18993670 includes a putative poly-A tail downstream of the probe, consistent with a mapping of A_23_P58137 to the ARL9 transcript, and thus, a truncated transcription termination site of *ARL9* in the UCSC genome browser.


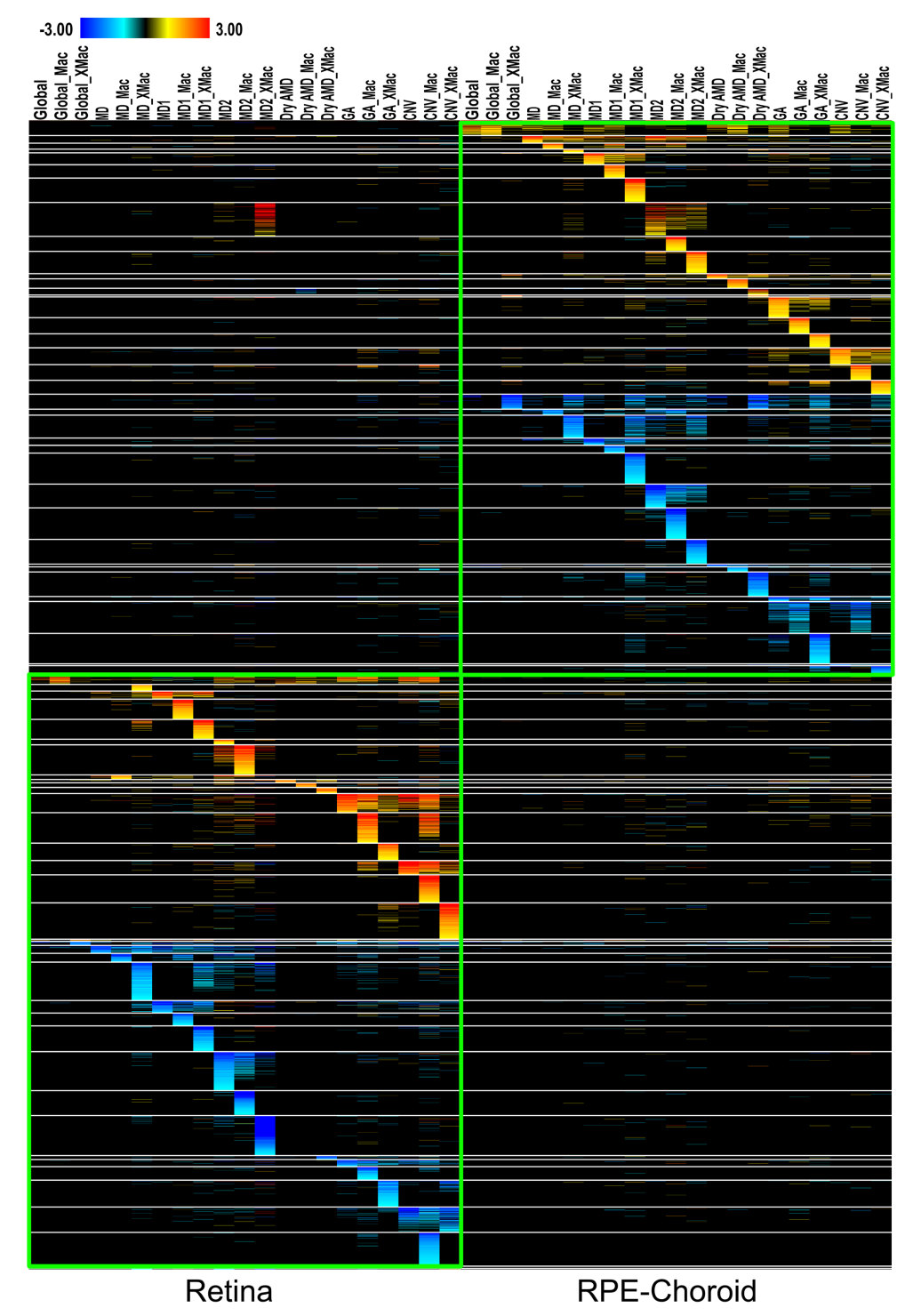


**Figure S6.** AutoSOME cluster results, related to Figure 2. Disease-associated genes with permuted p-values < 0.1 and fold difference > 1.5 were clustered based on their significance score (see *Materials and Methods*), and rendered as a heat map (color-scale identical to Figure 2). Columns represent disease classes (Retina on the left and RPE-choroid on the right) and rows are individual genes/probes. Individual clusters are separated by horizontal lines. Using cluster results inscribed within the top-right and bottom-left green rectangles, we identified “Disease Modules,” as shown in Figure 2 (see *Materials and Methods*). In some cases, minor manual adjustments were made to improve Disease Module quality. For example, we found clusters containing genes present in only GA or CNV, as well as present in both GA and CNV. In the latter case, genes were manually extracted into separate modules, called GA&CNV Up/Down (see Figure 2).

**
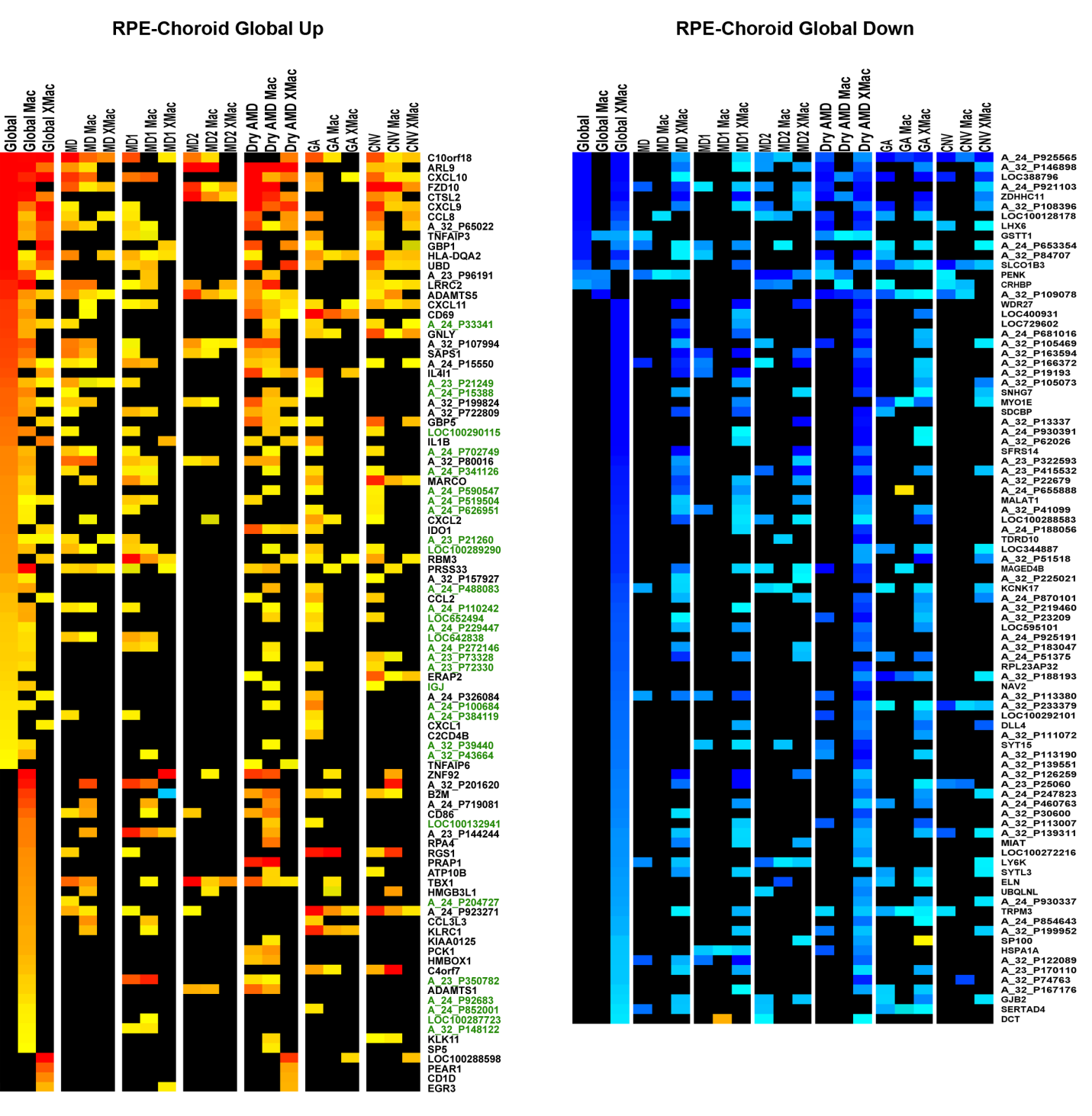
**

**Figure S7.** Full gene sets for RPE-choroid Global Up/Down Disease Modules, related to Figure 2a. The heat map color scale is identical to Figure 2. Green gene/probe symbols indicate immunoglobulins identified by gene expression clustering and confirmed by probe sequence alignment (see *Materials and Methods*), and are collapsed into a single gene, “Immunoglobulin,” in Figure 2a (right side), Figure 4b, and Figure S12.

**
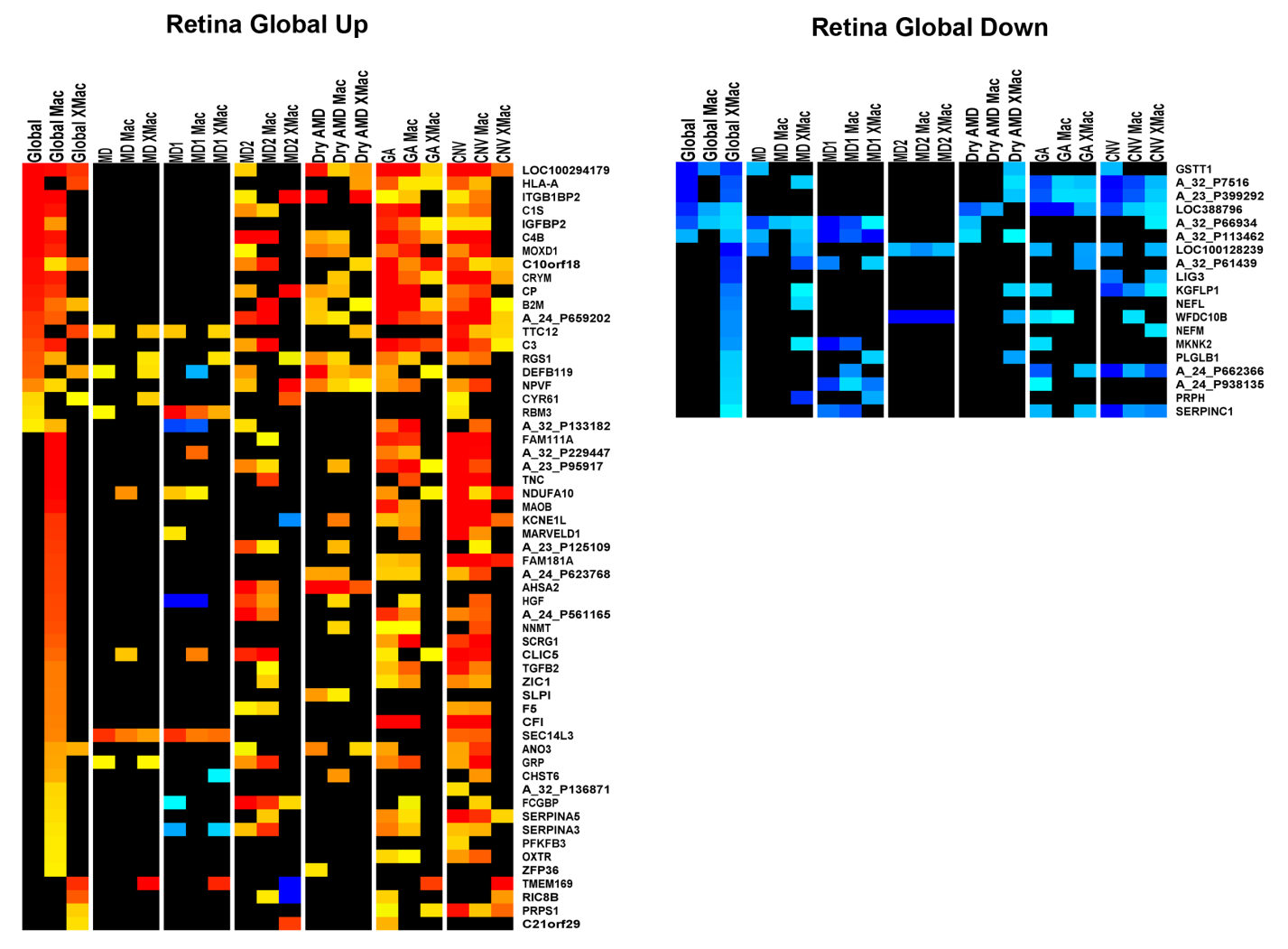
**

**Figure S8.** Full gene sets for retina Global Up/Down Disease Modules, related to Figure 2b. The color scale is identical to Figure 2.

**
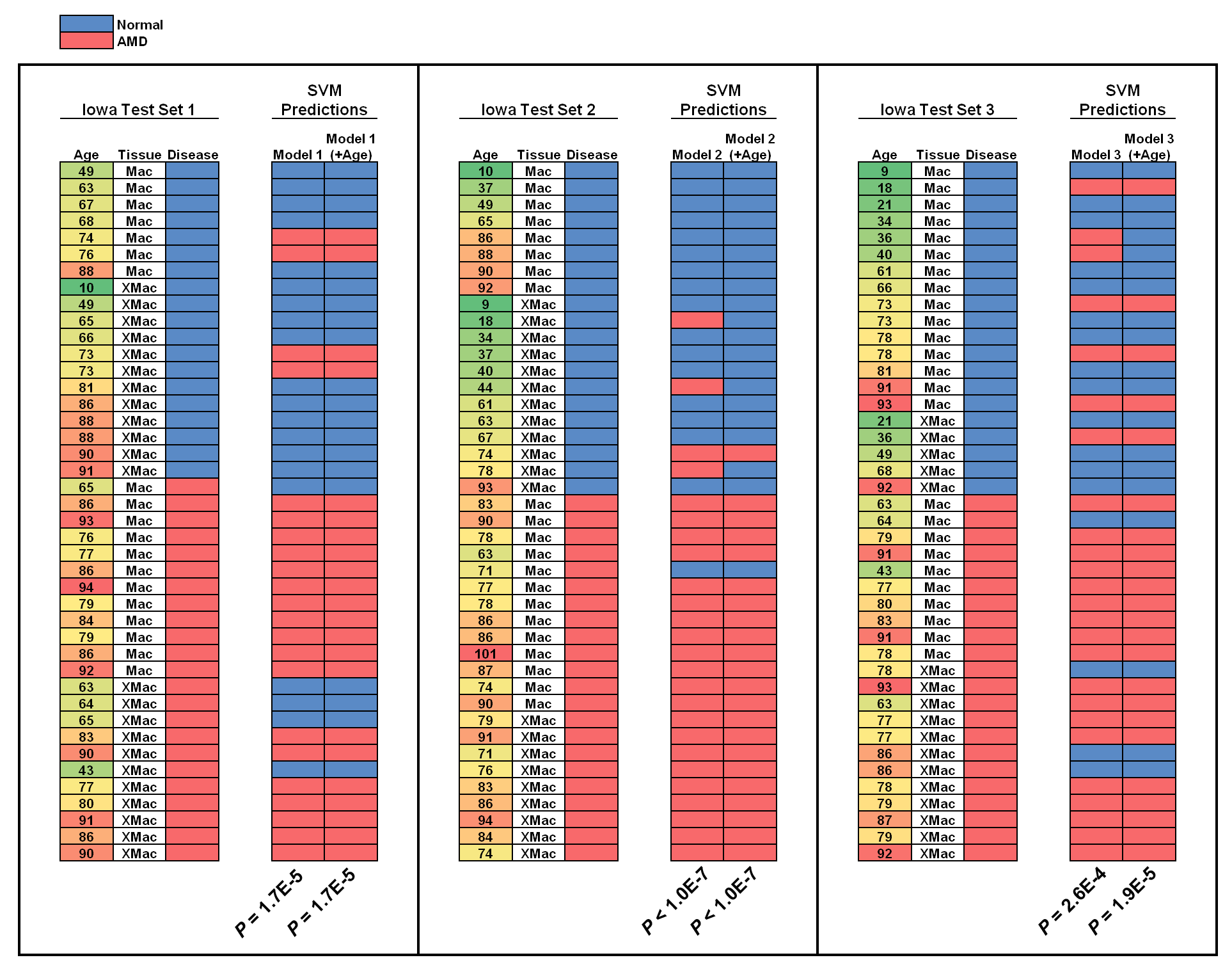
**

**Figure S9.** SVM results for three Iowa test datasets, related to Figure 3b. The full Iowa dataset (*n* = 126) was subdivided into three pairs of training and test datasets (see *Materials and Methods*). Using the expression profiles from the twenty genes shown in Figure 3a, each training set was used to build a separate SVM model, with and without age, and each SVM model was tested using the corresponding Iowa test dataset. The results are depicted as heat maps, with the statistical significance of each result shown underneath (determined by Monte Carlo sampling; see *Materials and Methods*). Mac = Macula; XMac = Extramacula.

**
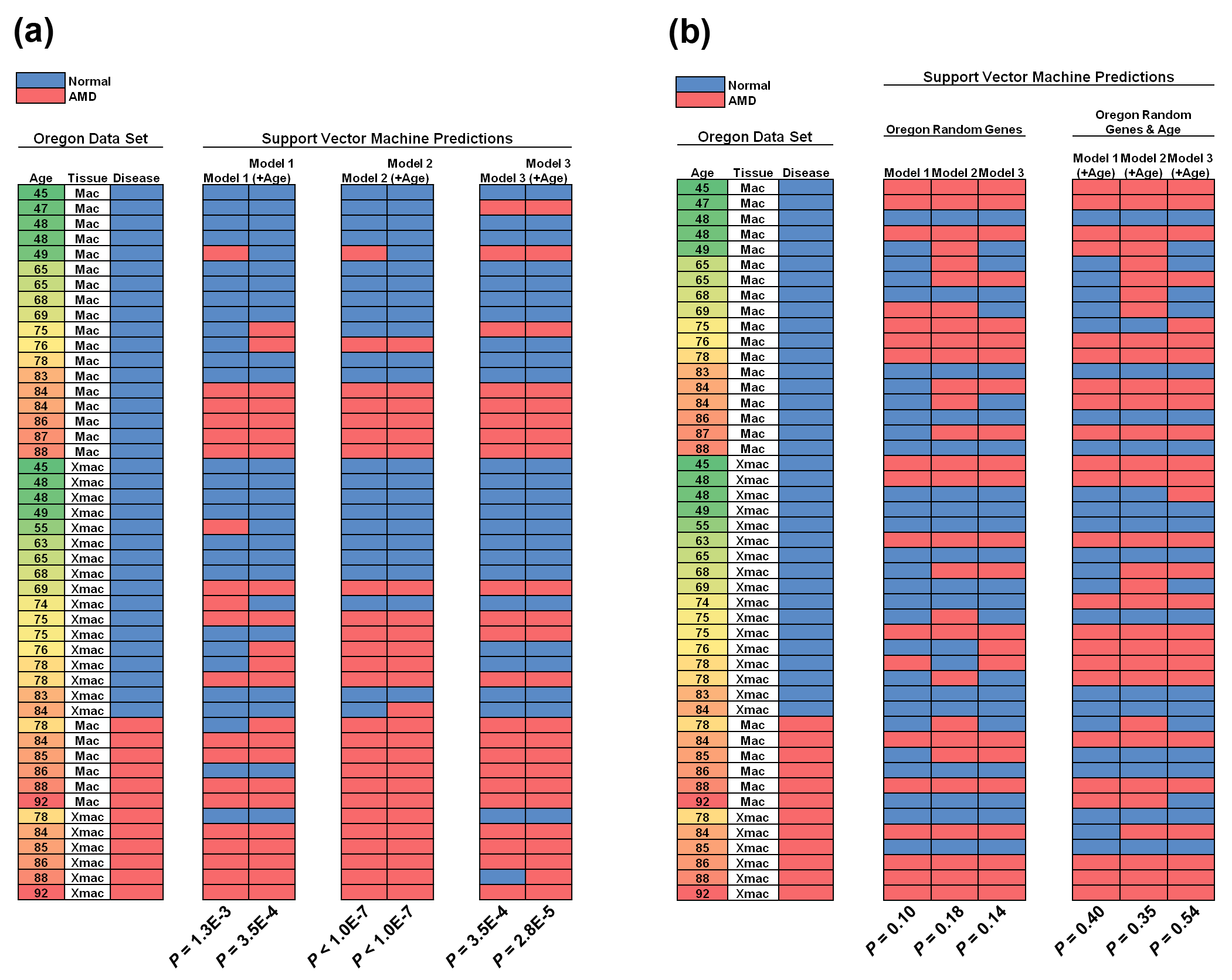
**

**Figure S10.** SVM results for Oregon expression data, related to Figure 3b. Results from applying each of three SVM models to the full Oregon dataset (**a**) and two random Oregon datasets (**b**) are depicted as heat maps, with the statistical significance of each result shown underneath (see *Materials and Methods*). Mac = Macula; XMac = Extramacula.

**
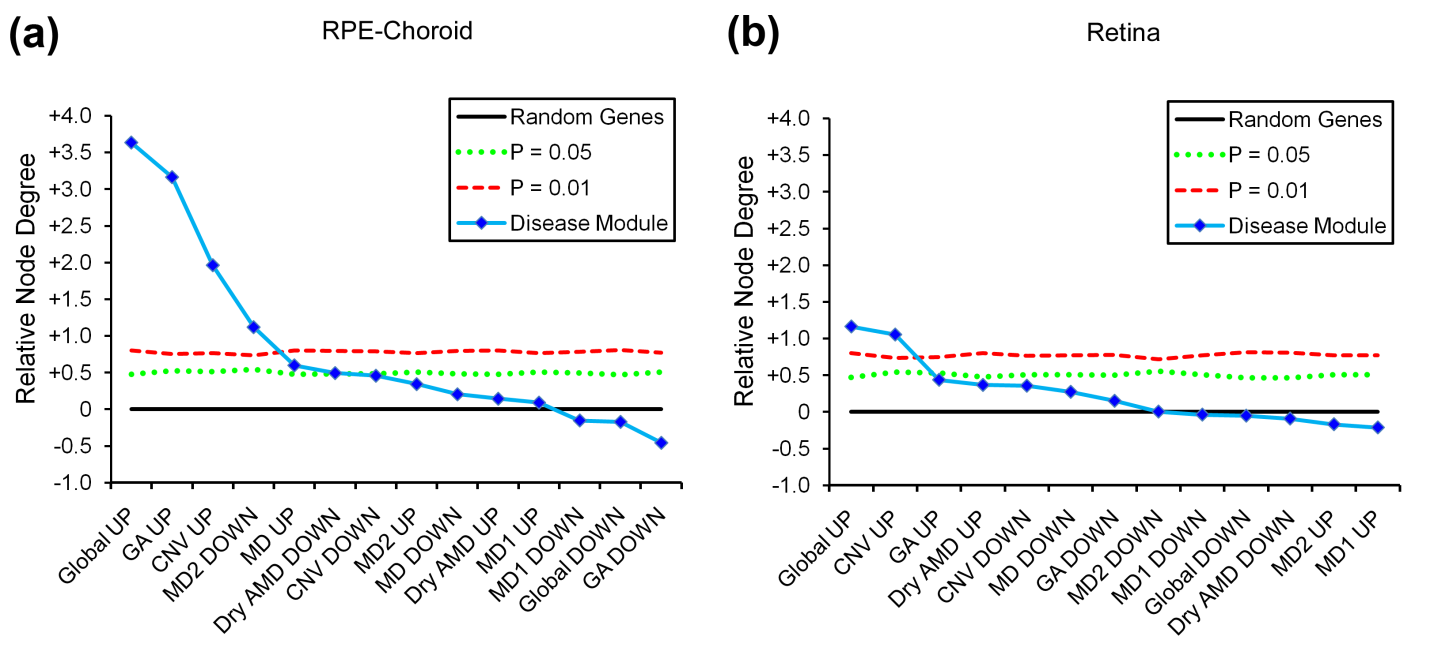
**

**Figure S11.** Statistical enrichment of protein-protein associations in each Disease Module. The statistical enrichment of protein-protein associations in each Disease Module is shown for (**a**) RPE-choroid and (**b**) retina datasets. All Disease Modules (see Figure 2) are shown on the x-axis, and ordered from the left by decreasing relative node degree (deviation in node degree from random chance). Node degree is equal to 2*No. edges divided by No. nodes, where edges are protein-protein interactions, and nodes correspond to proteins in the network. See *Materials and Methods* for further details.

**
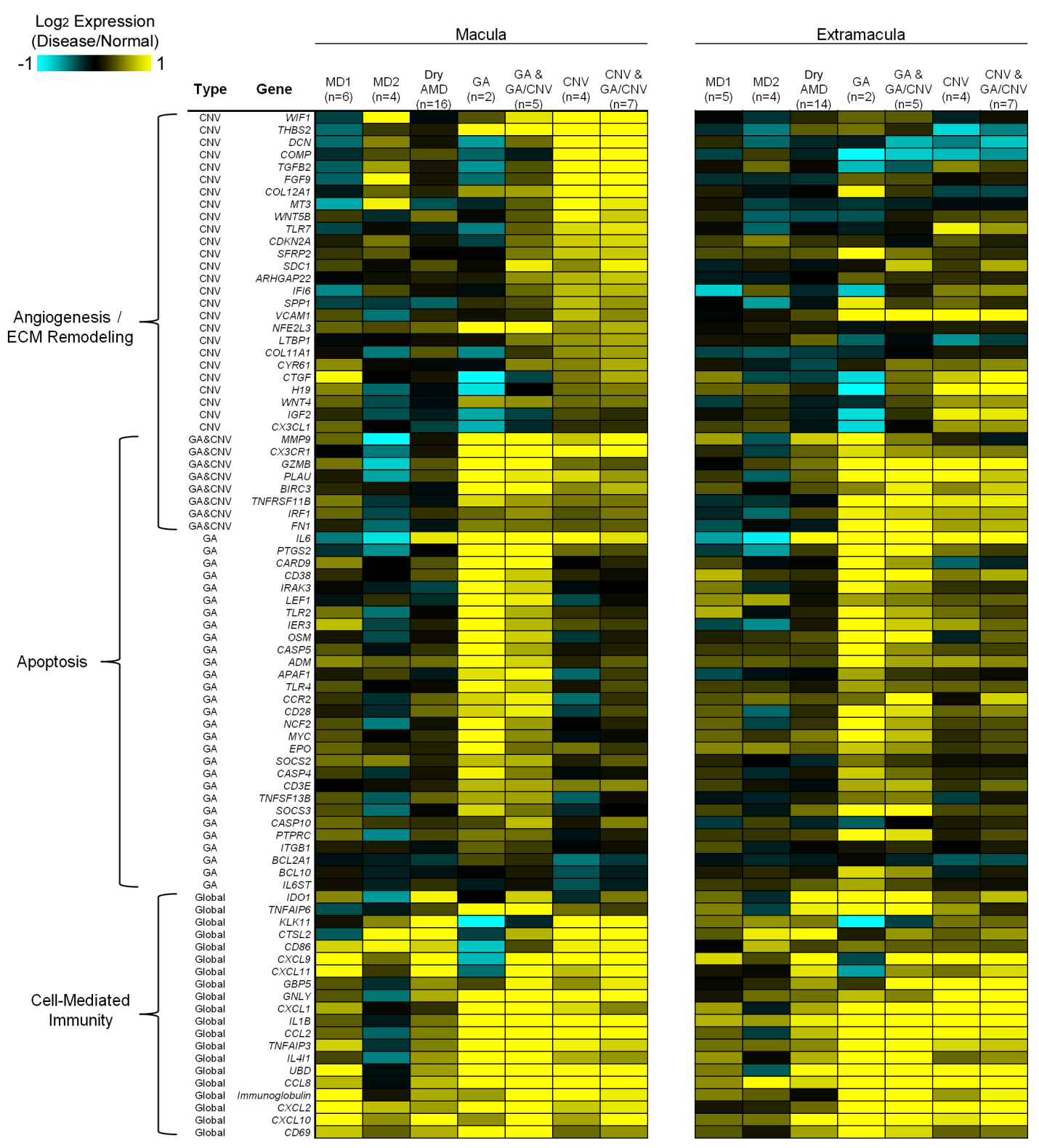
**

**Figure S12.** Heat map of normalized macular and extramacular expression levels for each gene in the RPE-choroid interactome depicted in Figure 4. Expression is normalized the same as described in Figure 4b. Genes are divided into the same three functional enrichment groups as in Figure 4. Columns represent donor sample classifications (see Table 1).


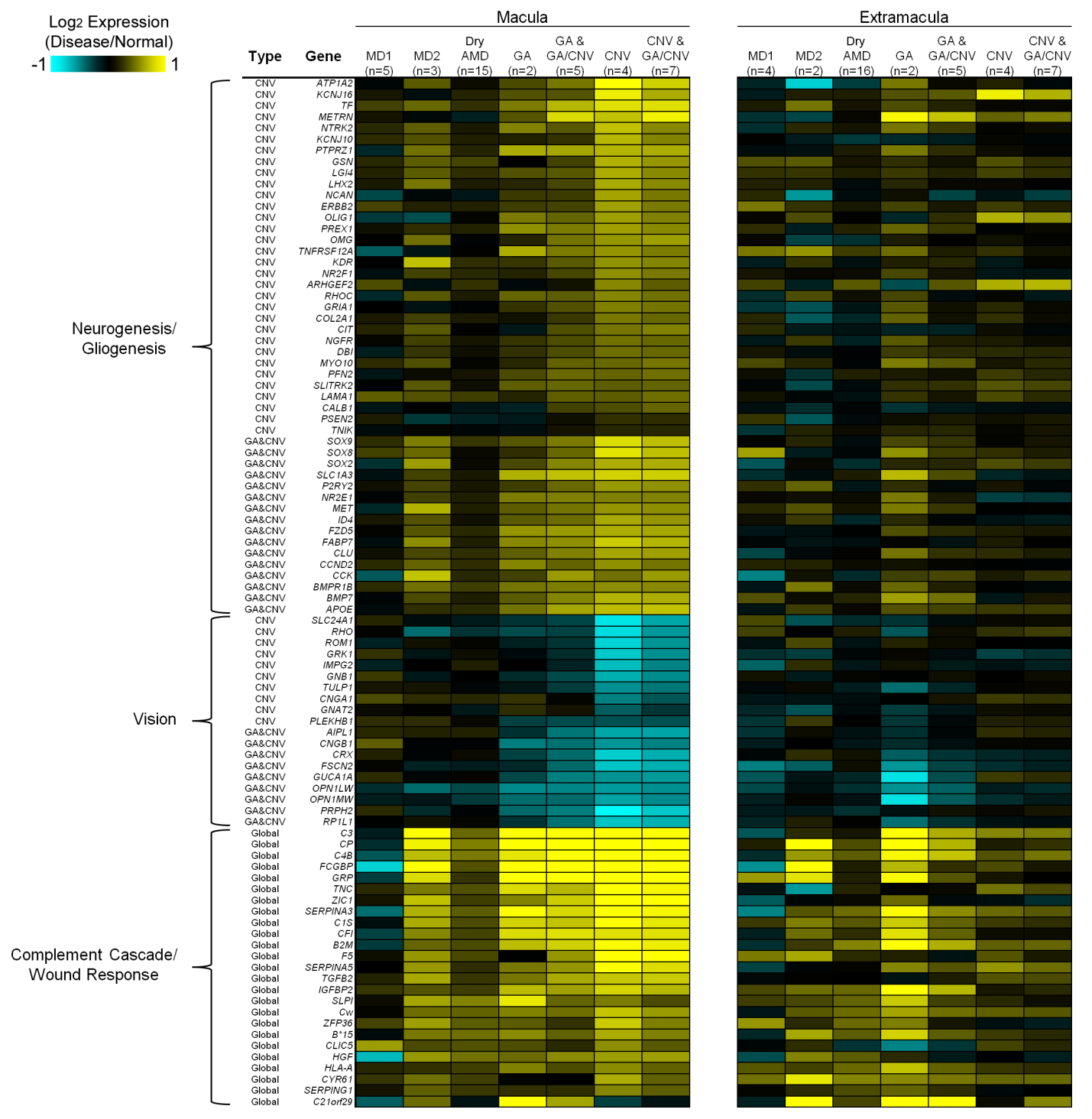


**Figure S13.** Heat map of normalized macular and extramacular expression levels for each gene in the retina interactome depicted in Figure 5. Expression is normalized the same as described in Figure 5b. Genes are divided into the same three functional enrichment groups as in Figure 5. Columns represent donor sample classifications (see Table 1).
