# Supplementary material for: Systems-level analysis of age-related macular degeneration reveals global biomarkers and phenotype-specific functional networks
Source: Genome Med. 2012 Feb 24;4(2):16. doi: 10.1186/gm315 (PMC3372225; doi:10.1186/gm315)
Supplement: Additional file 4 — Assessment of RNA and microarray quality. Description of analytical procedures used to determine the quality of the RNA and microarray data. [file gm315-S4.DOC]

**Systems-level analysis of age-related macular degeneration reveals global biomarkers and phenotype-specific functional networks**

Aaron M. Newman, Natasha B. Gallo, Lisa S. Hancox, Norma J. Miller, Carolyn M. Radeke, Michelle A. Maloney, James B. Cooper, Gregory S. Hageman, Don H. Anderson, Lincoln V. Johnson & Monte J. Radeke

**Assessment of RNA and Microarray Quality**

**RNA Quality**

As a general assessment of RNA quality, we analyzed a subset of the RPE-choroid and retina samples using microchannel electrophoresis. In total, 158 RNA samples were examined, and at least one RNA sample from 94 of 96 donors was analyzed. While all samples examined showed some degree of postmortem degradation, they all had distinct 18S and 28S ribosomal bands. For the entire dataset, the RNA Index Numbers (RIN) ranged from 4.0 to 8.7 electropherograms (mean = 6.7, standard deviation = 0.9), and representative RIN plots are shown in Additional file 1, Figure S2. All measured RINs are publicly available through the Gene Expression Omnibus [GEO: GSE29801]. As an additional assessment of RNA and microarray quality, we also examined the complete array set for evidence of increased noise as a function of RNA quality. The overall degree of inter-array differences for all RPE-choroid samples with a measurement of RIN was determined by calculation of the Pearson's correlation for all possible pairwise combinations. The results were ranked from most to least correlated and the resulting matrix was rendered as a heat map with the RIN of each sample displayed above (Additional file 1, Figure S3). As shown in Additional file 1, Figure S3, there is no clear association between RNA integrity and the value of the inter-array Pearson's correlation. Although the five samples with the worst RINs (< 5.0) were on the lower end of the correlation scale, they were also accompanied by many samples from the higher range of the integrity spectrum. Thus, within the range of RINs encountered with our samples, relative RNA integrity is not predictive of general array performance. However, this does not rule out that some transcripts may be sensitive to postmortem decay. When the Pearson's correlation between individual probes and RIN was determined for the variance-filtered Iowa RPE-choroid dataset (see *Materials and Methods* in the accompanying manuscript for dataset details), the intensity of 665 probes increased or decreased with increasing RIN using parametric p value < 0.001 (BRB Array Tools). Although significantly higher than that expected by random chance (~27 probes), only 65 probes had absolute correlation constants greater than 0.5 (absolute range of 0.24 - 0.61 for all 665 probes), and only 70 were found among the thousands of *Disease Module* genes listed in Additional file 5, Table S3. Gene ontology analysis [52] revealed enrichment for mitochondrial genes among the genes whose levels were higher in samples with more intact RNA and an increased level of contaminating retinal gene transcripts as a function of decreased RNA integrity. In conclusion, while differences in RNA integrity may have an impact on the analysis, the effect, if any, is minimal and likely only contributes to the overall noise in the data, thus reducing our ability to detect disease-related differences.

**Microarray Quality**

Additional analyses were performed to evaluate the Iowa and Oregon microarray datasets for inter-array concordance, agreement with previously published data, and overall data quality. For completeness, we included both normal and disease arrays in all quality-control exercises. To evaluate inter-array concordance, differentially expressed genes between RPE-choroid and retina were identified in the Iowa microarray dataset and subsequently compared to the same genes in the Oregon expression dataset using a heat map display. As shown in Additional file 1, Figure S4a, Oregon and Iowa RPE-choroid arrays display highly similar expression patterns for RPE-choroid enriched genes, indicating that both donor datasets capture equivalent aspects of RPE-choroid biology. Second, using Gene Set Enrichment Analysis (GSEA) [115], we analyzed the agreement between our expression data and publicly available transcriptome profiling experiments, and found highly significant enrichments for previously reported RPE-enriched genes [38] in Iowa/Oregon RPE-choroid samples (Additional file 1, Figure S4b, top panel) and for previously reported retina-specific genes [116] in Iowa retina samples (Additional file1, Figure S4b, lower panel). Next, we examined the expression patterns of 21 genes previously shown by qPCR and microarray analysis to exhibit differential expression between macula and extramacula samples in the RPE-choroid [36], and found comparable expression patterns in our combined Iowa/Oregon dataset (Additional file 1, Figure S4c). Finally, as a proxy for overall data quality, we examined expression of two gender-specific genes, *XIST* and *RPS4Y*, which are expressed in normal female and male human cells, respectively (e.g., Figure 5 in Day et al. [117]). As shown in Additional file 1, Figure S4d, these two genes are not only highly expressed in their corresponding samples across all 291 Iowa and Oregon arrays, but also show the expected mutually exclusive Boolean relationships. Taken together, these results indicate that both Iowa and Oregon microarray datasets are suitable for genomics analysis.
